# Geometry‐Encoded Actuation as a Structural Interaction Layer in Origami Robots

**DOI:** 10.1002/advs.76982

**Published:** 2026-08-14

**Authors:** Jianshu Zhou, Yitong Li, Junda Huang, Ian Zhang, Haoran Liu, Boyuan Liang, Yiyuan Zhang, Xiaohui Xiao, Qiguang He

**Affiliations:** ^1^ Department of Mechanical Engineering National University of Singapore Singapore; ^2^ Department of Mechanical and Automation Engineering The Chinese University of Hong Kong Hong Kong China; ^3^ Harvard John A. Paulson School of Engineering and Applied Sciences Harvard University Cambridge Massachusetts USA; ^4^ Department of Mechanical Engineering University of California Berkeley California USA; ^5^ School of Robotics Wuhan University Wuhan China

**Keywords:** geometry‐encoded actuation, multistable origami, structural interaction interface, stimuli‐responsive materials, soft robotics

## Abstract

Physical interaction in robotics predominantly relies on sensing, computation, and feedback control, resulting in increasing system complexity. Here, we introduce geometry‐encoded actuation (GEA), a design framework that exploits multistable origami geometries as programmable physical interaction layers to mechanically encode environmental stimuli into autonomous structural responses. By integrating multistable origami with liquid crystal elastomer (LCE) actuators, GEA couples structural geometry, energy landscapes, and stimuli‐responsive actuation to achieve programmable shape transformation without continuous sensing or complex control during the interaction process. A theoretical framework based on spherical geometry, energy analysis, and structural optimization is established to guide the design of bistable origami mechanisms. The proposed framework is validated through representative robotic systems, including a bistable origami gripper, an adaptive origami wheel, and autonomous locomotion and manipulation demonstrations, achieving up to 45% actuation strain, 106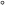 programmable geometric transformation, 100% task success in 10 consecutive trials, and stable operation after 500 actuation cycles. By embedding interaction intelligence directly into structural geometry, GEA redistributes part of the interaction complexity from computation to morphology, providing a general framework for programmable physical intelligence and adaptive embodied robotic systems.

## Graphical abstract

We present geometry‐encoded actuation (GEA), a design framework that integrates multistable origami with liquid crystal elastomer actuators to encode environmental interactions into autonomous structural responses. GEA enables programmable shape transformation without continuous sensing or complex control, achieving robust adaptive manipulation and locomotion through geometry‐driven physical intelligence.

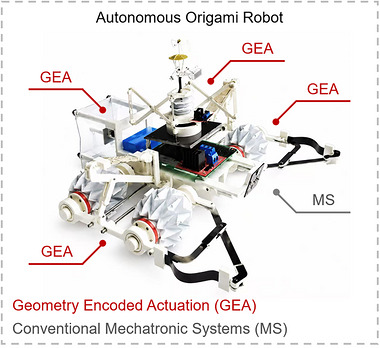

## 1 Introduction

Robotic systems increasingly operate in unstructured and dynamic environments, where physical interaction with the world is inevitable [1, 2]. Conventional robotic architectures interpret such interactions primarily through sensing and computation, relying on continuous perception, signal processing, and control loops to infer contact events and environmental states [3, 4, 5]. While effective, this paradigm tightly couples interaction understanding to sensing fidelity and algorithmic complexity, often leading to fragile performance, increased system overhead, and limited scalability in real‐world settings. Whether physical interaction itself can be encoded and processed at the structural level, rather than being entirely mediated by sensing and computation, remains an open and largely unexplored question [6, 7, 8].

Existing robotic systems typically treat mechanical structure as a passive substrate whose role is limited to force transmission and motion execution. Interaction is sensed externally, interpreted digitally, and regulated through control algorithms [9]. This separation between structure and interaction interpretation has driven rapid advances in perception and control, but at the cost of increasingly complex hardware–software stacks. In response, recent efforts in soft robotics [10, 11, 12, 13, 14] and embodied systems [15, 16, 17] have explored the use of compliant structures and stimuli‐responsive materials to embed aspects of sensing and response directly into the physical body. These approaches demonstrate that mechanical properties and material responses can simplify control and improve robustness [18, 19]. However, many such systems remain task‐specific, rely on continuous material deformation, or lack a unifying abstraction that generalizes across interaction modalities and tasks [20].

**FIGURE 1:**
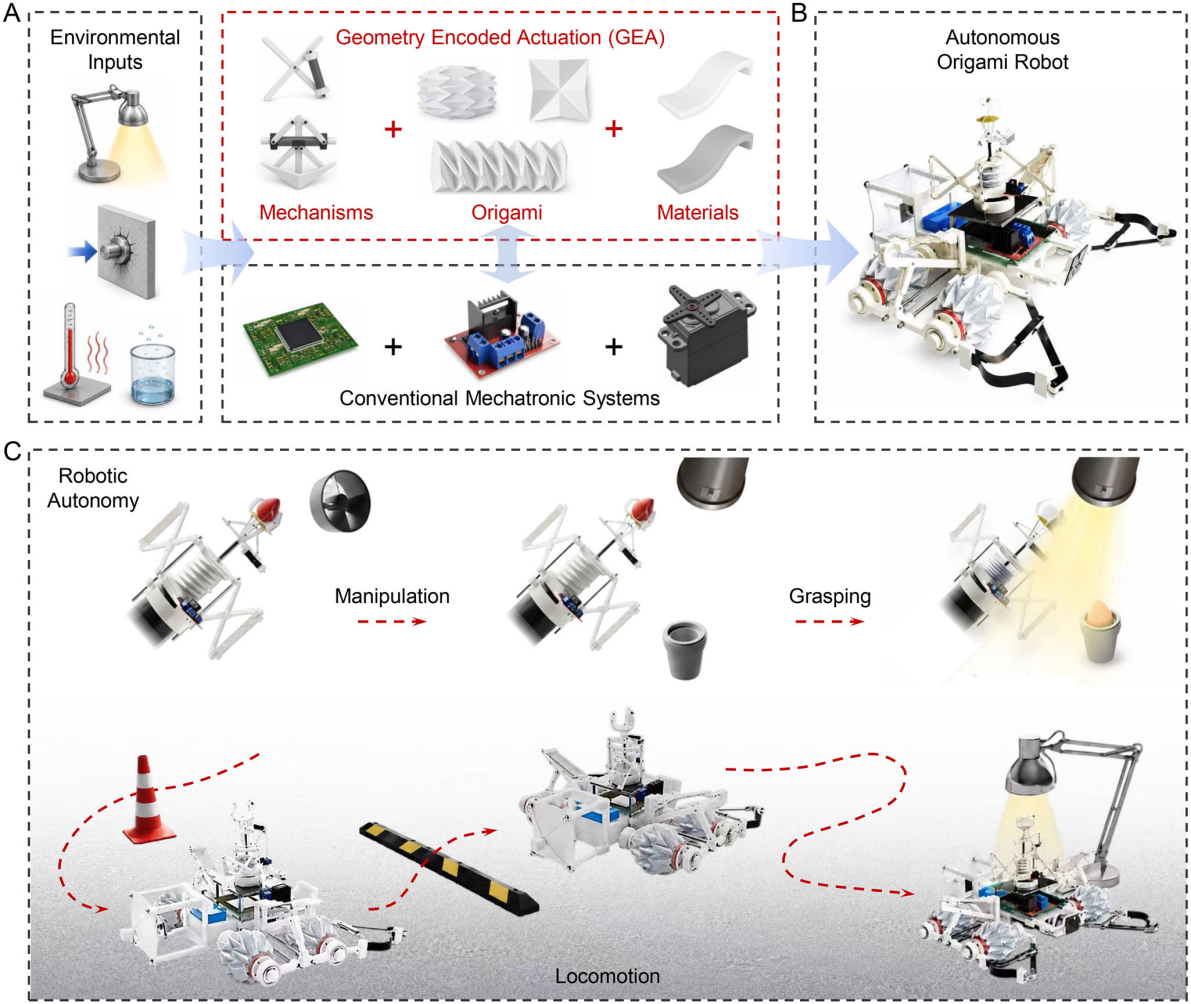
GEA as a design framework for robotic intelligence. (A) Conceptual framework of GEA, integrating environmental inputs, geometry‐programmed structures, conventional mechatronic systems (MS), and responsive materials. (B) Autonomous origami robot as an embodiment of the GEA framework, exhibiting stimulus‐responsive behavior through geometry‐encoded mechanisms. (C) Together, GEA, complemented by lightweight computational coordination, enables embodied origami robots capable of intelligent manipulation and locomotion.

Here, we propose a different perspective: rather than viewing structure as passive or considering intelligence to be material‐specific, we show that geometry itself can function as a physical interaction interface. Specifically, we introduce a geometry‐encoded actuation (GEA) framework, as shown in Figure 1A, in which structural geometry mediates robot–environment interaction by filtering, thresholding, and discretizing heterogeneous physical stimuli into stable, task‐relevant geometric state transitions. In this framework, interaction is not interpreted through continuous sensing, but is instead transformed directly by multistable geometric mechanisms into discrete structural states that can be reliably exploited by the system.

Origami‐inspired geometries provide a natural and powerful substrate for realizing this framework [21, 22, 23, 24, 25]. Due to their intrinsic kinematic constraints, geometric coupling, and multistability, origami mechanisms are well suited for encoding nonlinear interactions into discrete mechanical responses. In particular, bistable origami structures can act as mechanical thresholds [26, 27, 28, 29, 30], converting continuous or noisy inputs—such as contact forces, thermal stimuli, illumination, or moisture—into abrupt and repeatable state transitions. By coupling these geometries with stimuli‐responsive materials [31, 32], geometric state changes can be triggered both by direct physical contact and by contact‐free environmental stimuli, enabling a unified interaction abstraction across different modalities [33, 34].

In this work, we systematically investigate GEA mechanisms based on multistable origami geometries. We establish canonical geometric primitives that encode rotational and prismatic interactions through bistable transitions, and characterize how these structures transform low‐dimensional actuation inputs into functionally distinct configurations. Rather than optimizing for task‐specific performance, our focus is on identifying generalizable design principles that govern how geometry mediates interaction at the system level. Importantly, the same geometric abstraction applies regardless of whether state transitions are triggered by mechanical contact or by external stimuli, highlighting the role of geometry as a general interaction interface.

To validate the generality and practical relevance of this framework, we implement GEA in representative manipulation and locomotion systems (Videos S1 and S2). As shown in Figure 1B, an autonomous origami robot integrates multistable origami structures, transmission mechanisms, and stimuli‐responsive actuators to achieve adaptive behaviors through physically encoded state transitions. Rather than relying on continuous sensing and feedback control, environmental stimuli directly trigger geometric reconfiguration, enabling responsive interactions at the structural level. As illustrated in Figure 1C, GEA combined with lightweight computational coordination supports a range of intelligent robotic functions in both manipulation and locomotion. Across both systems, structural geometry mediates interaction by transforming environmental inputs into discrete, task‐relevant state transitions, while computation remains responsible for high‐level coordination and sequencing.

Together, these results position structural geometry as an effective interaction layer between robots and the physical world. By redistributing interaction complexity from sensing and computation to morphology, the GEA framework provides a strategy with the potential to be adapted to diverse robotic systems. Rather than treating interaction solely as a signal to be sensed and processed, this perspective demonstrates how geometric structure can directly encode and regulate environmental response at the physical level, complementing existing material‐driven and algorithmic approaches.

## 2 Results and Discussion

### 2.1 Geometry‐Encoded Actuation (GEA) Mechanism

**FIGURE 2:**
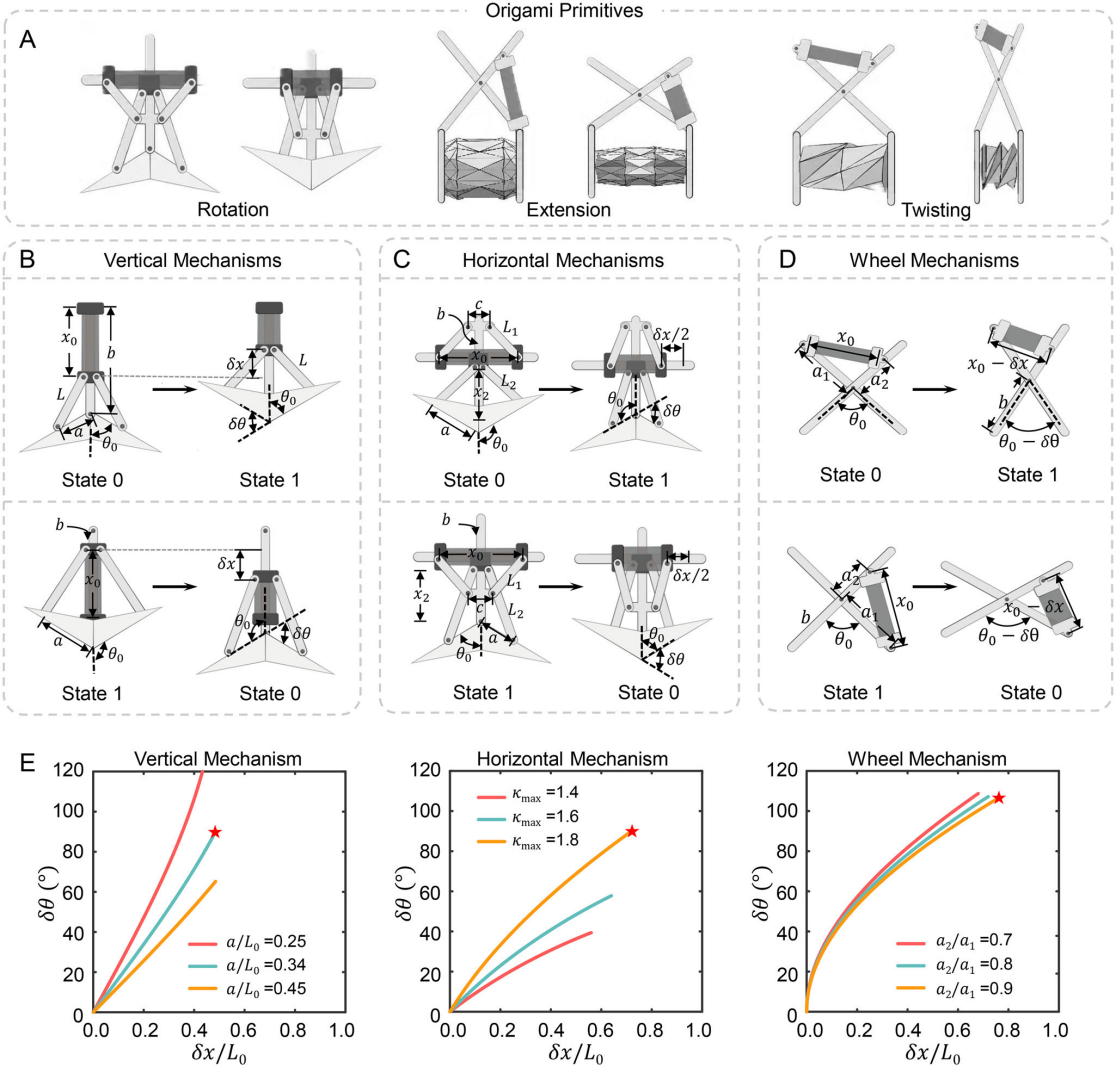
GEA mechanism design and normalized LCE stroke–rotation curves. (A) Three origami motion primitives. (B) Vertical mechanisms actuated by heating source between two geometric states. (C) Horizontal mechanisms actuated by light source between two geometric states. (D) Wheel mechanisms with both horizontal and vertical LCE installations: water‐triggered actuation from state 0 to state 1 showing dry state LCE and actuated state LCE, and light‐triggered actuation from state 1 to state 0. (E) Geometry‐derived stroke–rotation design maps with SQP‐optimized design points. Red markers denote the selected optimal design points.

In this work, we establish a GEA framework that generates three fundamental origami motion primitives: rotation, extension, and twisting, as illustrated in Figure 2A.

The rotation primitive is realized through a bistable origami mechanism integrated with transmission linkages and an liquid crystal elastomer (LCE) actuator. Depending on the installation orientation of the LCE, two configurations, termed the vertical and horizontal mechanisms, are obtained. Upon thermal stimulation, the reversible contraction and extension of the LCE drive the origami structure to transition between its two stable states (state 0 and state 1), thereby producing a discrete rotational motion.

The extension and twisting primitives are generated by a wheel mechanism composed of a Yoshimura origami structure, transmission linkages, and an LCE actuator. By reconfiguring the orientation of the embedded LCE, the same structural architecture can be programmed to undergo either axial extension or torsional deformation, yielding distinct geometric outputs from a common actuation principle.

Rather than relying on conventional motor‐driven control, the GEA mechanism exploits the intrinsic shape‐morphing capability of LCEs [35, 36] to encode and trigger geometric state transitions directly within the structure. Through the co‐design of origami geometry, linkage topology, and responsive material actuation, motion generation is embedded into the physical architecture itself, enabling programmable mechanical behaviors without complex sensing or control systems.

#### 2.1.1 Vertical and Horizontal Mechanisms

The design and geometric parameters of the vertical and horizontal mechanisms are presented in Figure 2B,C. The fundamental distinction between the vertical and horizontal mechanisms lies in the installation orientation of the LCE, which is arranged vertically and horizontally, respectively. Despite this difference, both mechanisms share the same basic architecture, consisting of an LCE actuator, transmission linkages, and an origami mechanism.

Depending on the angular configuration, the origami mechanism exhibits two discrete geometric states, denoted as state 0 and state 1, corresponding to negative and positive angular configurations, respectively. Both the vertical and horizontal mechanisms include two design variants: one in which state 0 serves as the initial configuration and state 1 as the actuated configuration, and another in which state 1 serves as the initial configuration and state 0 as the actuated configuration. To facilitate unified kinematic and static optimization, all four mechanism variants are described using the same set of geometric and mechanical parameters. Considering the maximum length change of the LCE is 40%, we hope to achieve a large revolute motion with a small LCE length change.

##### Design Goal and Decision Variables

Given the maximum contraction ratio of the LCE (40%), we aim to achieve a large revolute motion of the origami flap with a small relative LCE length change. We formulate the design as a nonlinear constrained optimization problem solved by sequential quadratic programming (SQP). The decision variables are defined in a unified manner as the LCE rest length $x_{0}$, the LCE length change δx, the slide length b, the distance between the fixed point and the origami center a, the transmission lengths L, $L_{1}$, and $L_{2}$, and the origami rotation angle change δθ. Here, L is used for the vertical mechanisms, whereas $L_{1}$ and $L_{2}$ are used for the horizontal mechanisms.

We aim to minimize the objective function

(1) $$F_{obj}=w_{1}{\left(\frac{\delta x}{x_{0}}\right)}^{2}+w_{2}\;b^{2}-w_{3}\;{\left(\delta \theta \right)}^{2}-w_{4}a^{2},$$

where $w_{1},w_{2},w_{3},w_{4}>0$ are scalar weights. The term $\frac{\delta x}{x_{0}}$ means we hope the LCE changes its length as small as possible. The term b means we need the vertical slide to be short. The term δθ means we hope the origami flap can be as large as possible. The term a means we need the fixed point to move away from the origami center.

The four mechanism variants satisfy three categories of constraints. The first category consists of length constraints. For the vertical mechanisms, the length of the vertical sliding guide must exceed the length of the LCE, the maximum contraction strain of the LCE is limited to 40%, and the transmission slide is subject to a geometric length constraint. Accordingly, the following conditions are imposed:

(2) $$0<x_{0}\leq b,\;0<\delta x\leq 0.4x_{0},\;0<L\leq L_{0}.$$

For the horizontal mechanisms, the horizontal slide and the two connecting rods form a triangular configuration to avoid torque singularity. Similarly, the maximum contraction strain of the LCE is limited to 40%, and the transmission links are constrained by

(3) $$0<x_{0}<2L_{1},\;0<\delta x\leq 0.4x_{0},\;0<L_{1},L_{2}\leq L_{0}.$$

The second category consists of angular constraints. For mechanisms whose initial configuration corresponds to state “0,” the angular relationship is given by

(4) $$2\theta_{0}+\delta \theta =\pi .$$

For mechanisms whose initial configuration corresponds to state “1,” the corresponding constraint becomes

(5) $$\delta \theta +\pi =2\theta_{0}.$$

The third category consists of geometric constraints derived from the law of cosines. For the vertical mechanism with state “0” as the initial configuration, the geometric relationships are

(6) $$\begin{array}{cc} L^{2} & =a^{2}+d_{1}^{2}-2ad_{1}\cos\left(\theta_{0}\right), \end{array}$$

(7) $$\begin{array}{cc} L^{2} & =a^{2}+d_{2}^{2}+2ad_{2}\cos\left(\theta_{0}\right), \end{array}$$

where $d_{1}=b-x_{0}+\delta x,\;d_{2}=b-x_{0}$.

For the vertical mechanism with state “1” as the initial configuration, the geometric constraints are

(8) $$\begin{array}{cc} L^{2} & =a^{2}+x_{0}^{2}+2ax_{0}\cos\left(\theta_{0}\right), \end{array}$$

(9) $$\begin{array}{cc} L^{2} & =a^{2}+{\left(x_{0}-\delta x\right)}^{2}+2a\left(x_{0}-\delta x\right)\cos\left(\theta_{0}-\delta \theta \right). \end{array}$$

For the horizontal mechanism with state “0” as the initial configuration, the geometric relationships are

(10) $$\begin{array}{cc} L_{2}^{2} & =a^{2}+b^{2}-2ab\cos\left(\theta_{0}\right), \end{array}$$

(11) $$\begin{array}{cc} L_{2}^{2} & =a^{2}+d_{3}^{2}+2ad_{3}\cos\left(\theta_{0}\right), \end{array}$$

where $d_{3}=b-\sqrt{L_{1}^{2}-{\left(\frac{x_{0}-\delta x}{2}\right)}^{2}}+\sqrt{L_{1}^{2}-{\left(\frac{x_{0}}{2}\right)}^{2}}$.

For the horizontal mechanism with state “1” as the initial configuration, the geometric constraints are

(12) $$\begin{array}{cc} L_{2}^{2} & =a^{2}+d_{4}^{2}+2ad_{4}\cos\left(\theta_{0}\right), \end{array}$$

(13) $$\begin{array}{cc} L_{2}^{2} & =a^{2}+d_{5}^{2}+2ad_{5}\cos\left(\theta_{0}-\delta \theta \right), \end{array}$$

where $d_{4}=b-\sqrt{L_{1}^{2}-{\left(\frac{x_{0}}{2}\right)}^{2}},\;d_{5}=b-\sqrt{L_{1}^{2}-{\left(\frac{x_{0}-\delta x}{2}\right)}^{2}}$.

For horizontal mechanisms, we further consider the practical requirement that the geometric quantities $d_{3},d_{4}$, and $d_{5}$ remain non‐negative. To prevent the formation of a degenerate triangular configuration, an additional clearance constraint is imposed

(14) $$0<x_{0}\leq \kappa_{\max}L_{1},\;\kappa_{\max}<2.$$

For the vertical mechanisms, reduction of the geometric constraints reveals that both mechanism variants satisfy the same stroke–rotation relationship,

(15) $$\delta x=2a\sin\left(\frac{\delta \theta }{2}\right).$$

However, the original weighted objective function admits degenerate near‐singular solutions and therefore cannot, by itself, provide a physically meaningful and unique optimum. To obtain a practical design, three additional engineering considerations are imposed: the LCE is assumed to operate at its maximum allowable contraction, the vertical slide is minimized to reduce the overall size, and the transmission length reaches its packaging limit. These conditions can be expressed as $\delta x=0.4x_{0},\;b=x_{0},\;L=L_{0}$.

Under these assumptions, closed‐form optimal solutions can be derived for both mechanism variants. For a target angular displacement of $\delta \theta^{*}=90^{\circ }$, the optimized design parameters of the vertical mechanisms are obtained as

(16) $$a^{*}=\frac{L_{0}}{\sqrt{1+15\sin^{2}\left(\delta \theta /2\right)}},\;\delta x^{*}=\frac{2L_{0}\sin\left(\delta \theta /2\right)}{\sqrt{1+15\sin^{2}\left(\delta \theta /2\right)}}.$$

Substituting $\delta \theta^{*}=90^{\circ }$ yields $\frac{a^{*}}{L_{0}}\approx 0.3430,\;\frac{\delta x^{*}}{L_{0}}\approx 0.4851$.

For the horizontal mechanisms, similar assumptions are adopted. Specifically, the LCE is assumed to operate at its maximum allowable contraction, and the first connecting rod is taken to reach its admissible upper bound, namely, $\delta x=0.4x_{0},\;L_{1}=L_{0}$.

For the target angular displacement $\delta \theta^{*}=90^{\circ }$ and the design margin $\kappa_{\max}=1.8$, the common optimal design variables for the horizontal mechanisms are obtained as

(17) $$x_{0}^{*}=1.8L_{0},\;\delta x^{*}=0.72L_{0},\;a^{*}=0.2869L_{0}.$$

The remaining geometric parameters are determined by enforcing the shortest feasible slide condition. Specifically, $d_{3}^{*}=0$ and $d_{5}^{*}=0$ are imposed for the 0→1 and 1→0 horizontal mechanisms, respectively. Finally, the resulting normalized optimal design parameters for all four mechanism variants can be summarized in Table 1.

**TABLE 1:** Normalized optimal design parameters of the four mechanism variants.

Mechanism	$a^{*}/L_{0}$	$\delta x^{*}/L_{0}$	$b^{*}/L_{0}$	$\delta \theta^{*}$
Vertical (0→1, 1→0)	0.3430	0.4851	1.2128	$90^{\circ }$
Horizontal (0→1)	0.2869	0.7200	0.4058	$90^{\circ }$
Horizontal (1→0)	0.2869	0.7200	0.8417	$90^{\circ }$

#### 2.1.2 Wheel Mechanism

The wheel based on the Yoshimura origami pattern is capable of bistable transformation, enabling reversible changes in its axial length. The transition between the two stable states has been realized experimentally, as shown in Figure 2D. Similar to the previous mechanisms, the design optimization is formulated as a SQP problem.

##### Design Goal and Decision Variables

The decision variables are defined as b, δx, $a_{1}$, $a_{2}$, $x_{0}$, and δθ. Here, b, δx, $x_{0}$, and δθ retain the same definitions as in the previous section, while $a_{1}$ and $a_{2}$ denote the distances from the two LCE attachment points to the intermediate hinge of the transmission linkage, respectively. Without loss of generality, the attachment point on one side of the LCE is assumed to be closer to the hinge than the other, yielding $a_{2}<a_{1}$. The optimization objective that needs to be minimized is defined as

(18) $$F_{\mathrm{obj},w}=w_{1}{\left(\frac{\delta x}{x_{0}}\right)}^{2}+w_{2}b^{2}-w_{3}{\left(\delta \theta \right)}^{2}-w_{4}a_{2}^{2}.$$

The optimization is subject to several geometric constraints. First, the initial length of the LCE must remain positive, and the maximum contraction strain of the LCE is limited to 40%. Therefore,

(19) $$0<\delta x<0.4x_{0}.$$

Furthermore, the wheel is assumed to be installed in a horizontal configuration, which imposes the geometric relationship

(20) $$b\cdot \sin\left(\frac{\theta_{0}}{2}\right)=\frac{L_{0}}{2}.$$

For the transition from state 0 to state 1, the angle becomes larger, therefore an additional constraint is

(21) δθ<0.

For the transition case from state 1 to state 0, two triangle constraints are

(22) $$\begin{array}{c} a_{1}^{2}+a_{2}^{2}-2a_{1}a_{2}\cos\left(\theta_{0}\right)=x_{0}^{2}, \end{array}$$

(23) $$\begin{array}{c} a_{1}^{2}+a_{2}^{2}-2a_{1}a_{2}\cos\left(\theta_{0}-\delta \theta \right)={\left(x_{0}-\delta x\right)}^{2}, \end{array}$$

while for the transition case from state 0 to state 1, the constraints are

(24) $$\begin{array}{cc} a_{1}^{2}+a_{2}^{2}-2a_{1}a_{2}\cos\left(\pi -\theta_{0}\right)= & x_{0}^{2}, \end{array}$$

(25) $$\begin{array}{cc} a_{1}^{2}+a_{2}^{2}-2a_{1}a_{2}\cos\left(\pi -\theta_{0}+\delta \theta \right)= & {\left(x_{0}-\delta x\right)}^{2}. \end{array}$$

Using finite packaging constraints, optimized attachment lengths are obtained at active bounds, giving $a_{1}^{*}=L_{0}$ and $a_{2}^{*}=0.9L_{0}$. For the transition from state 1 to state 0, the optimization yields $\theta_{0}^{*}=\pi$, which corresponds to the minimum wheel‐axis length $b^{*}=L_{0}/2$. The associated LCE rest length is $x_{0}^{*}=a_{1}^{*}+a_{2}^{*}=1.9L_{0}$, and the maximum admissible contraction therefore becomes $\delta x^{*}=0.4x_{0}^{*}=0.76L_{0}$. Under these parameters, the resulting angular transformation is $\delta \theta^{*}=106.47^{\circ }$. These results indicate that the 1→0 wheel mechanism can realize a large rotational transition while maintaining a compact axial installation length.

By contrast, for the transition from state 0 to state 1, no unique numerical optimum is obtained when δθ is treated as a free optimization variable. Instead, the feasible solutions form a one‐dimensional trade‐off curve: increasing $\theta_{0}$ shortens the wheel‐axis length b, but simultaneously reduces the achievable magnitude of the angular transformation |δθ|.

Furthermore, to provide a more intuitive interpretation of the geometric analysis and optimization results, the stroke–rotation relationships predicted by the geometric model are illustrated in Figure 2E. The curves describe the dependence of the achievable origami rotation change δθ on the LCE length change δx and are not optimization objective functions. The marked optimal points correspond to the solutions of the constrained SQP optimization problem. Owing to the imposed geometric and engineering constraints, the optimal solutions are found on the feasible boundaries and therefore coincide with the peak points of the corresponding curves.

### 2.2 Experiments

We conduct experiments to characterize both the actuator and the origami mechanical structure, with a particular focus on how temperature and water affect reaction time and strain.

#### 2.2.1 Material—LCE Characterization

**FIGURE 3:**
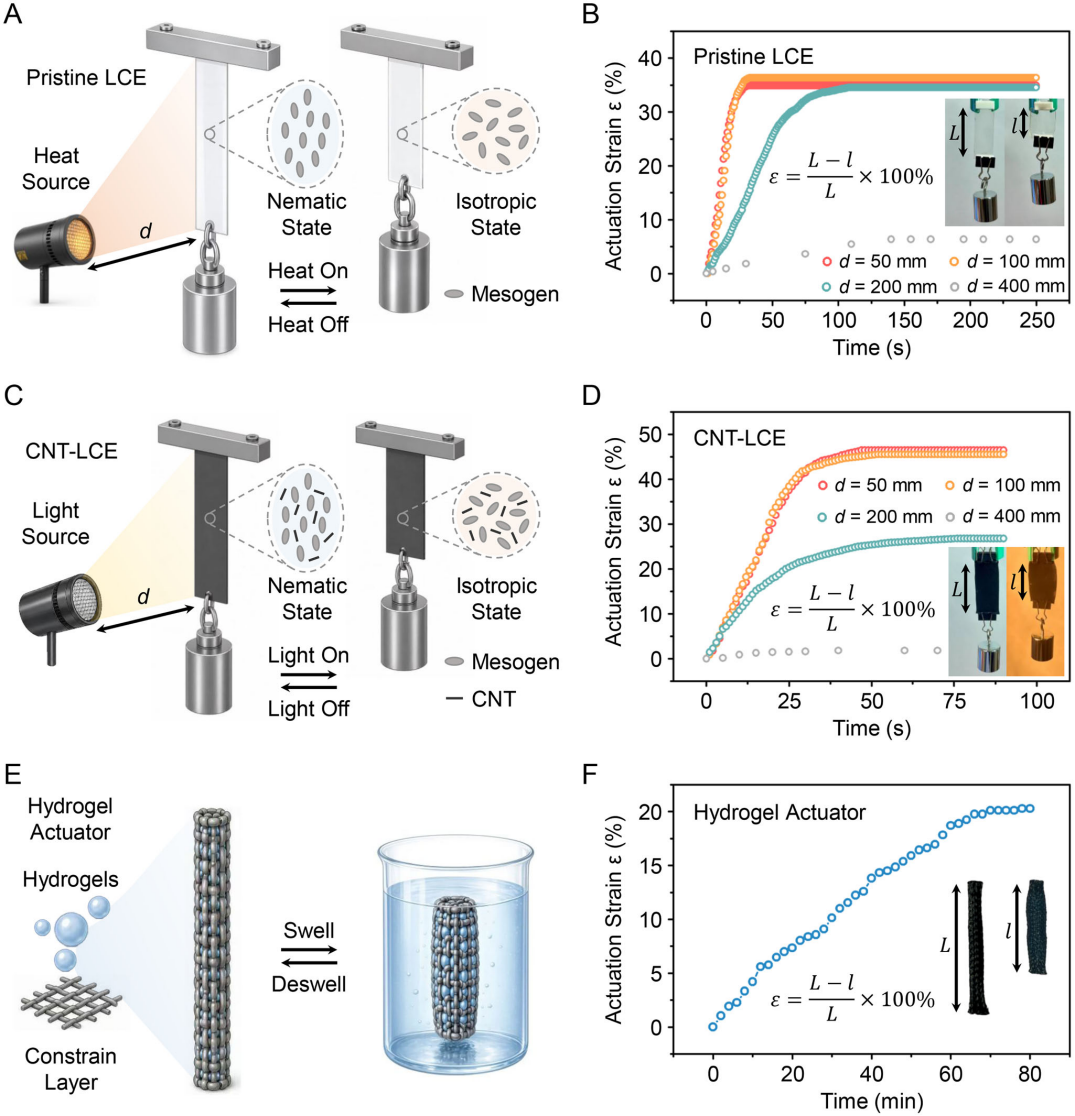
Working principles and actuation performances of different types of actuators. (A) Schematic of a pristine lLCE actuator reversibly lifting a weight under a heat source, driven by the phase transition of liquid crystal molecules from an ordered to a disordered state. (B) Actuation strain as a function of time for the pristine LCE actuator at various distances from the heat source. (C) Schematic of a carbon nanotube‐LCE (CNT‐LCE) photothermal actuator reversibly lifting a weight under a light source, transitioning from an ordered to a disordered molecular alignment. (D) Actuation strain as a function of time for the CNT‐LCE actuator at various distances from the light source. (E) Schematic of a hydrogel‐based actuator undergoing reversible contraction through swelling and deswelling behaviors in response to water. (F) Actuation strain profile as a function of time for the hydrogel actuator during the swelling process.

We characterize LCEs in terms of heating distance, temperature, and actuation strain. Two types of LCEs are used: transparent and black. Both are fabricated using the same base materials and procedures, with the black variant incorporating an additional pigment [37, 38]. The initial states of the LCEs are shown in Figure 3A,C. Upon heating, the LCE temperature increases and contraction is observed.

As shown in Figure 3A, a 200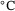 heating source is positioned at a variable distance d from the transparent LCE. Two key observations are made from Figure S4: (1) the maximum stable temperature depends on the distance beyond 50 mm, the temperature stabilizes below 200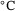; and (2) shorter distances result in faster temperature rise. From the temperature–time and strain–time plots in Figure 3B, we find that (1) the maximum actuation strain is approximately 37%, and (2) a distinct contraction‐point temperature exists. Beyond this temperature threshold, further heating primarily influences the actuation speed rather than the magnitude.

For the black LCE, a 200 W light source is used (Figure 3C), with d again representing the distance to the LCE. The maximum achievable temperature is around 100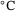, with significant reductions in stable temperature at distances greater than 100 mm. The strain–time plot (Figure 3D) shows a maximum strain of 45%. A contraction‐point temperature is also identified, beyond which temperature mainly affects the actuation speed.

The responsive material in the proposed GEA framework is not limited to LCEs and can be replaced by other stimuli‐responsive actuators. As an example, a hydrogel‐based linear actuator is illustrated in Figure 3E. Through its swelling and deswelling behaviors in response to water absorption and release, the actuator can achieve reversible contraction and extension. The corresponding actuation strain evolution is shown in Figure 3F. Similar to LCEs, the hydrogel actuator exhibits a stable and reversible deformation process; however, its actuation strain changes more gradually due to the relatively slower diffusion‐controlled response mechanism. These results highlight the material‐agnostic nature of the GEA framework, which can be integrated with a wide range of responsive materials while preserving the same actuation principle.

#### 2.2.2 Structure–Origami Characterization

The proposed origami mechanisms are validated in this section. As discussed earlier, six configurations of origami mechanisms are studied (Figure 2B–D) [39, 40]. By exploiting the bistable nature of square and wheel‐type origami structures, we achieve discrete state transitions through linear actuation by LCE actuators.

**FIGURE 4:**
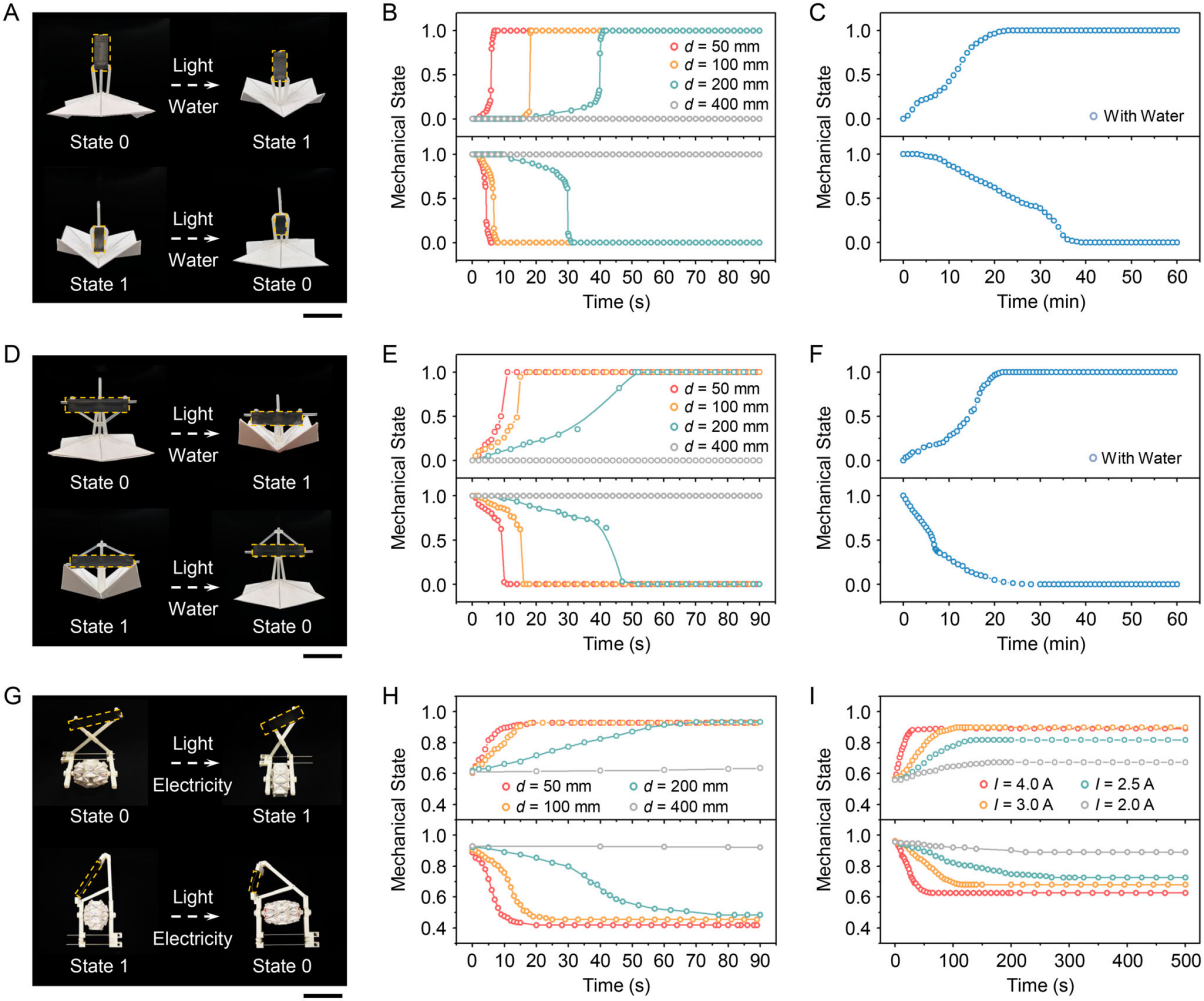
Stimulus‐responsive GEA mechanisms and their state‐transition behaviors. d denotes the distance between the light source and the LCE, and I denotes the heating current. Scale bars in A, D: 30 mm, scale bar in G: 50 mm. (A) Vertical mechanisms actuation under light or water stimuli. (B) State‐transition responses of vertical mechanisms under light stimulation at different d. (C) State‐transition responses of vertical mechanisms under water stimulation. (D) Horizontal mechanisms actuation under light or water stimuli. (E) State‐transition responses of horizontal mechanisms under light stimulation at different d. (F) State‐transition responses of horizontal mechanisms under water stimulation. (G) Wheel mechanisms actuation under light or electrical stimuli. (H) State‐transition responses of wheel mechanisms under light stimulation at different d. (I) State‐transition responses of wheel mechanisms under electrical stimulation at different I.

Figure 4A shows the setup where the LCE is mounted perpendicularly to the origami. Transitions between states 0 and 1 are achieved by adjusting the heating distance d (Figure 4B). A smaller d results in faster heating and quicker state transitions.

Similarly, in the parallel configuration (Figure 4D), the LCE is mounted along the plane of the origami. Although the actuation mode is similar to the perpendicular configuration, indirect stress propagation leads to energy absorption by the structure, resulting in smoother state transitions (Figure 4E).

Compared to LCEs, hydrogel‐driven actuation exhibits slower transitions, and the smooth strain curve indicates a more gradual stress response (Figure 4C,F).

The origami wheel employs a scissor mechanism and exhibits bistability between its shortest and longest lengths, as shown in Figure 4G. We define a normalized state index as $\frac{l_{\max}-l}{l_{\max}-l_{\min}}$. The light source distance d and heating current I are critical parameters. Effective actuation occurs when the contraction‐point temperature is reached, as presented in Figure 4H,I.

### 2.3 System‐Level Demonstrations

We present two full‐system demonstrations that showcase the implementation of GEA in origami robots for contact‐based and contact‐free interaction. The first case is an reactive manipulation system, the second case is a reactive locomotion system.

#### 2.3.1 Manipulation

**FIGURE 5:**
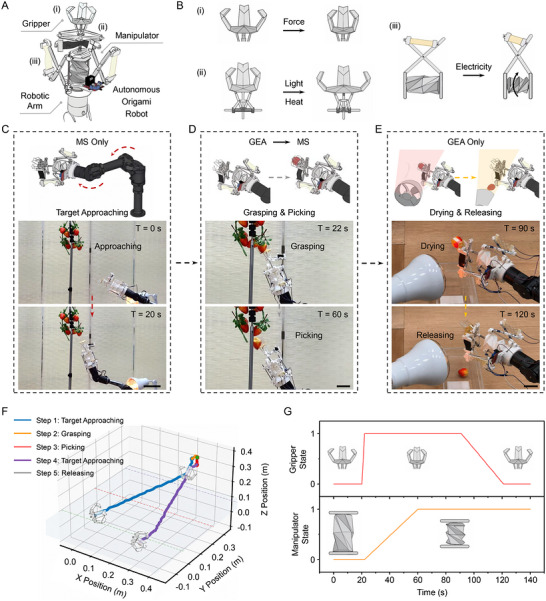
GEA enabled origami‐based dexterous end‐effector for autonomous fruit manipulation. Scale bars in C, D, E: 80 mm. (A) Schematic of the environmentally responsive origami end‐effector, consisting of a bistable gripper and a dual‐Kresling wrist. (B) State transitions of the gripper and wrist modules. (C) Target‐approaching stage of the fruit pick‐and‐place process. (D) Grasping and picking stage, where contact triggers gripper closure and wrist twisting. (E) Releasing stage, where external heating or illumination triggers fruit release at the target location. (F) End‐effector trajectory during a complete manipulation cycle. (G) State‐transition responses of the gripper and wrist during the fruit pick‐and‐place process.

To demonstrate the implementation of GEA in manipulation, we developed an origami‐based dexterous end‐effector consisting of a bistable gripper and a dual‐Kresling wrist (Figure 5A) [41]. The electrical system, powered by an 11.1 V Li‐ion battery, drives the heating coils embedded in the transparent LCEs. This design leverages three functional layers: (i) intrinsic geometric logic from the origami bistable mechanism, (ii) stimulus‐responsive actuation from black and transparent LCEs, and (iii) mechatronic systems (MS) for perception, timing, and high‐level coordination. Together, these create an embodied manipulation system capable of multi‐stage, context‐dependent behaviors.

**FIGURE 6:**
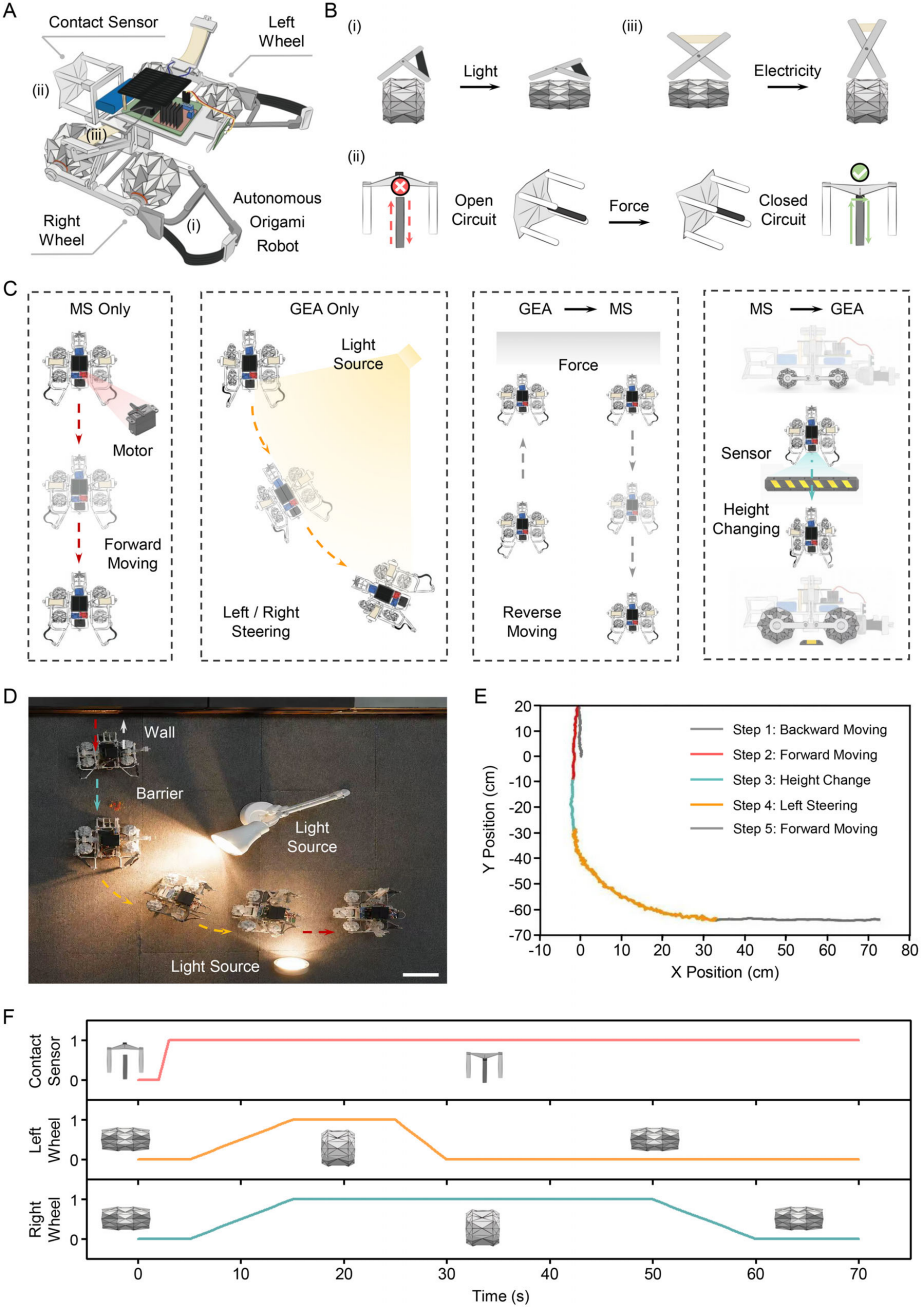
GEA enabled origami vehicle for autonomous and adaptive locomotion. Scale bar in D: 150 mm. (A) Schematic of the environmentally responsive origami vehicle featuring a multifunctional wheel mechanism and a bistable origami trigger. (B) Origami mechanisms enabling adaptive locomotion: (i) light‐actuated wheel mechanism, (ii) waterbomb‐based triggering sequence, and (iii) electrically actuated wheel mechanism. (C) Interaction architecture integrating GEA with perception, inference, and motor control during locomotion. (D) Representative locomotion sequence demonstrating autonomous obstacle negotiation, directional switching, and steering behaviors. (E) Vehicle trajectory during autonomous locomotion. Different colors indicate distinct locomotion stages. (F) State‐transition responses of the origami mechanisms during locomotion.

The gripper is constructed from the bistable structure characterized in Figure 2. A central bistable cell anchors three finger flaps cut from the surrounding sheet, forming a cage‐like grasping mechanism. The bistable base enables two complementary actuation modes. In the extrinsic mode, a physical push transitions the system from state 1 to 0, identical in principle to the origami trigger in Figure 6B. In the intrinsic mode, the black LCE contracts under heating or illumination, pulling the bistable mechanism from state 0 to 1 and thereby reopening the gripper. This dual‐mode bistability allows the end‐effector to autonomously respond to contact‐based forces and contact‐free stimuli.

The wrist consists of two Kresling origami units connected in series. Each Kresling module exhibits coupled axial contraction and rotational twist upon compression. By stacking two units, the wrist achieves amplified contraction and twisting motion. Two symmetric transparent LCE actuators power the wrist: when activated, they contract and drive the Kresling pair to simultaneously shorten and rotate, generating a pulling‐and‐twisting action that facilitates fruit detachment.

Leveraging this hybrid structure, the dexterous end‐effector demonstrates two distinct interaction paradigms, illustrated in Figure 5C–E:

- *Contact‐based*: During reaching, physical contact with the fruit snaps the bistable mechanism to its closed state, generating a stable grasp. This mechanical transition also closes the embedded circuit, sending a GEA‐generated trigger to the MS module. The controller responds by heating the transparent LCE actuators, causing the wrist to contract and twist to detach the fruit.
- *Contact‐free*: During the drying phase, the black LCE acts simultaneously as a timer and a trigger. When exposed to a controlled electrical heating or lighting source, it contracts at a calibrated rate. After a preset period, the contraction drives the bistable gripper from state 0 to 1, autonomously releasing the fruit without requiring sensing, timing loops, or computation.

The complete fruit‐picking sequence is shown in Figure S7. First, the arm approaches the fruit under algorithmic control (i). Upon contact, the gripper closes automatically through contact‐based interaction (ii). The closed state triggers the transparent LCEs, contracting and twisting the wrist to detach the fruit (iii). The end‐effector then transports the fruit to the drying area for GEA‐driven release through the black LCE (iv). A three‐dimensional trajectory of the end‐effector (Figure 5F) highlights the transitions between contact‐based trigger and contact‐free picking. As shown in Figure 5G, both the gripper and wrist manipulator undergo a sequence of state transitions over time, demonstrating their coordinated operation during the fruit pick‐and‐place task.

Overall, this manipulation demonstration illustrates how multistable geometry structures can encode physical contact and stimuli‐responsive actuation into task‐relevant state transitions. When combined with algorithmic control, the system can perform complex tasks such as reaching, grasping, detaching, and transporting.

#### 2.3.2 Locomotion

Achieving autonomous locomotion in origami robots is challenging because their actuation strokes, material nonlinearities, and sensing capabilities are inherently constrained. Traditional systems typically rely either on electronic subsystems featuring continuous sensing and control or external stimuli that may lead to inconsistent motion. To overcome this separation, we developed an untethered origami car that integrates geometry‐encoded origami triggers and stimuli‐responsive LCEs, together with algorithmic perception, inference, and motor control (Figure 6A). This co‐design reduces the need for continuous sensing during geometry‐mediated interactions while enabling rapid responses to trajectory changes.

The platform incorporates transparent and black LCE actuators, a bistable origami trigger, an onboard camera, and an Arduino Nano‐based controller. Each component contributes a distinct yet interdependent role. The transparent LCE contracts under Joule heating to raise the wheel height, as shown in Figure 6B‐iii, facilitating body elevation for obstacle traversal. The black LCE contracts under external light, producing asymmetric deformation that generates passive steering without requiring computation. As shown in Figure 6B‐ii, the origami trigger consists of a square bistable pattern with an embedded conductive magnet. The initial convex state corresponds to an open circuit; a mechanical push flips it into its concave state, closing the circuit and sending a discrete electrical signal to the microcontroller. This bistable switching enables reliable physical‐to‐electrical transduction without continuous power. Together, these mechanisms create a combined contact‐based and contact‐free interaction platform, which can navigate through obstacles and respond to environmental stimuli (Figure 1).

Leveraging this architecture, the car demonstrates four locomotion behaviors, illustrated in Figure 6C:

- *Behavior 1: Contact‐free Navigation*. The robot moves forward on origami wheels under algorithmic control. Using the onboard camera, the MS module continuously monitors obstacles within a 30–50 cm range and maintains heading.
- *Behavior 2: Contact‐free Steering*. When illuminated by external light exceeding 25 mW/${\mathrm{cm}}^{2}$, the black LCE contracts by approximately 2.3 mm within 1–2 s, generating a turning angle of about 12
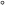
–16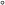 per 10 cm of travel. This passive, light‐driven steering does not require perception, computation, or control.
- *Behavior 3: Contact‐free Height Change*. When the camera detects an obstacle taller than roughly 20 mm, the MS controller activates the transparent LCE to raise the body by ∼4.8 mm in approximately 3 s. This height increase allows the origami car to climb over the obstacle, demonstrating MS‐triggered physical reconfiguration.
- *Behavior 4: Contact‐based Reversal*. Upon collision with an object, the origami trigger snaps from its convex to concave bistable state, closing the electrical circuit. This contact‐based interaction provides a robust, high‐signal‐to‐noise mechanical event that triggers an immediate directional change.

The complete locomotion sequence is shown in Figure 6D,E. The robot first advances under algorithmic control (Step 1). Upon encountering a wall at approximately x=15 cm, the bistable trigger flips under contact‐based interaction and initiates reversal command (Step 2). As it moves backward, the camera detects a second obstacle, prompting a contact‐free height‐change process that enables traversal (Step 3). After clearing the obstacle, two external light sources induce contact‐free turning, producing a cumulative rotation of roughly 35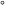 and establishing a new forward heading (Steps 4 and 5). The recorded trajectory quantitatively reflects these transitions and highlights how contact‐based and contact‐free interactions dynamically alternate in governing locomotion. Figure 6F presents the state‐transition responses of the contact sensor, left wheel, and right wheel during locomotion process.

To evaluate the long‐term mechanical durability of the proposed origami structures, fatigue characterization experiments were conducted on the key mechanisms used in both system‐level demonstrations. As summarized in Tables S1 and S2, both the waterbomb origami gripper and the origami wheel mechanism maintained stable functional operation over 500 continuous actuation cycles. The gripper preserved reliable bistable switching behavior, while the wheel mechanism sustained repeatable expansion– contraction functionality, despite gradual and localized softening of crease regions during extended operation. These results provide an initial assessment of the fatigue behavior of the origami components under repeated actuation. Further improvements in material selection and fabrication processes may further enhance long‐term durability.

Moreover, to assess repeatability, both the manipulation and locomotion demonstrations were repeated for 10 consecutive trials under typical laboratory conditions. All trials successfully completed the intended tasks, yielding a success rate of 100%. These results indicate that the proposed GEA framework provides consistent performance under the tested operating conditions, although further validation in more diverse and long‐term scenarios remains an important direction for future work. Overall, this demonstration reveals that GEA can be leveraged for multi‐stage, adaptive locomotion. Noisy or abrupt environmental stimuli can be filtered and encoded by geometry‐encoded structures, which facilitates smooth and consistent locomotion in complex, rapidly changing scenarios.

## 3 Conclusion and Future Work

In this work, we introduced GEA, a design framework that exploits multistable origami geometries as programmable physical interaction layers for robotic systems. Rather than relying exclusively on continuous sensing, computation, and feedback control, GEA directly transforms heterogeneous environmental stimuli into discrete structural state transitions through geometry‐encoded mechanisms. By integrating multistable origami with stimuli‐responsive actuators, the proposed framework establishes a unified strategy for autonomous physical interaction across both contact‐based and contact‐free scenarios, providing a geometry‐centric approach to physical intelligence.

To realize this concept, we established a unified theoretical framework based on spherical geometry, geometric constraints, energy analysis, and structural optimization to guide the design of bistable origami mechanisms. The resulting framework generates three fundamental motion primitives, including rotation, extension, and twisting, through programmable geometric transitions. Representative robotic systems, including a bistable origami gripper and an adaptive origami wheel, experimentally validated the proposed framework, achieving up to 45% actuation strain, 106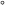 programmable geometric transformation, 100% task success over repeated 10 trials, and stable operation after 500 continuous actuation cycles. These results demonstrate that complex physical interactions can be regulated directly through structural geometry without continuous sensing during the interaction process.

Beyond the demonstrated robotic platforms, GEA provides a broader perspective on embodied robotic intelligence. Instead of treating mechanical structures as passive components for force transmission, the proposed framework enables structural geometry to actively encode, filter, threshold, and regulate interactions with the environment. Consequently, part of the interaction complexity is redistributed from sensing and computation to morphology, while high‐level computation remains responsible for task planning and coordination. This complementary relationship between geometry and computation offers a scalable strategy for simplifying robotic systems while maintaining robust and adaptive behaviors in uncertain environments. More importantly, because geometric encoding is locally embedded within each multistable mechanism, the framework naturally supports modular system design and can be extended to more complex robotic assemblies.

Future work will focus on extending the GEA framework along several directions. First, richer multistable geometries beyond binary switching will be explored to expand geometric encoding from bistable to multistable or continuously discretized structural state spaces, enabling more expressive and programmable physical interactions. Second, the framework will be integrated with a broader range of stimuli‐responsive materials, including electroactive, magnetic, and chemically responsive polymers, to expand the spectrum of environmental stimuli that can be directly encoded into structural responses. Third, quantitative co‐design methodologies that simultaneously optimize structural mechanics, material actuation, and interaction behaviors will be developed. Such analytical and computational models are expected to systematically characterize the trade‐offs among stability, switching thresholds, response speed, and energy efficiency, providing a rigorous foundation for geometry‐mediated interaction. Finally, hierarchical integration of multiple geometry‐encoded modules into larger robotic systems may enable distributed physical intelligence across different spatial and functional scales, allowing local geometric state transitions to naturally interface with high‐level planning and coordination. Overall, this work establishes GEA as a general design framework for embedding physical intelligence into robotic structures, providing a scalable route toward adaptive robotic systems in which interaction is encoded directly by morphology rather than continuously reconstructed through sensing and control.

## Conflicts of Interest

The authors declare no conflicts of interest.

## Supporting information


**Supporting File 1**: advs76982‐sup‐0001‐SuppMat.pdf.

**Supporting File 2**: advs76982‐sup‐0002‐VideoS1.mp4.

**Supporting File 3**: advs76982‐sup‐0003‐VideoS2.mp4.

## Data Availability

The data that support the findings of this study are available from the corresponding author upon reasonable request.

## References

[1] A. Billard and D. Kragic , “Trends and Challenges in Robot Manipulation,” Science 364, no. 6446 (2019): eaat8414, 10.1126/science.aat8414. 31221831

[2] D. Rus and M. T. Tolley , “Design, Fabrication and Control of Soft Robots,” Nature 521, no. 7553 (2015): 467–475, 10.1038/nature14543. 26017446

[3] C. C. de Wit , B. Siciliano , and G. Bastin, eds., Theory of Robot Control (Springer Science & Business Media, 2012).

[4] K.‐S. Fu , R. C. Gonzalez , C. S. G. Lee , and H. Freeman , Robotics: Control, Sensing, Vision, and Intelligence (McGraw‐Hill, 1987).

[5] B. Siciliano and O. Khatib , “Robotics and the Handbook,” in Springer Handbook of Robotics (Springer International Publishing, 2016), 1–6.

[6] A. Alú , A. F. Arrieta , E. D. Dottore , et al., “Roadmap on Embodying Mechano‐Intelligence and Computing in Functional Materials and Structures,” Smart Materials and Structures 34, no. 6 (2025): 063501, 10.1088/1361-665X/ad3f4f.

[7] M. Sitti , “Physical Intelligence as a New Paradigm,” Extreme Mechanics Letters 46 (2021): 101340, 10.1016/j.eml.2021.101340. 35475112 PMC7612657

[8] H. Liu , D. Guo , and A. Cangelosi , “Embodied Intelligence: A Synergy of Morphology, Action, Perception and Learning,” ACM Computing Surveys 57, no. 7 (2025): 1–36, 10.1145/3696898.

[9] J. Wang and A. Chortos , “Control Strategies for Soft Robot Systems,” Advanced Intelligent Systems 4, no. 5 (2022): 2100165, 10.1002/aisy.202100165.

[10] C. Lee , M. Kim , Y. J. Kim , et al., “Soft Robot Review,” International Journal of Control, Automation and Systems 15, no. 1 (2017): 3–15.

[11] E. W. Hawkes , L. H. Blumenschein , J. D. Greer , and A. M. Okamura , “A Soft Robot That Navigates Its Environment Through Growth,” Science Robotics 2, no. 8 (2017): eaan3028, 10.1126/scirobotics.aan3028. 33157883

[12] M. Calisti , G. Picardi , and C. Laschi , “Fundamentals of Soft Robot Locomotion,” Journal of the Royal Society Interface 14, no. 130 (2017): 20170101, 10.1098/rsif.2017.0101. 28539483 PMC5454300

[13] K. Becker , C. Teeple , N. Charles , et al., “Active Entanglement Enables Stochastic, Topological Grasping,” Proceedings of the National Academy of Sciences 119, no. 42 (2022): e2209819119, 10.1073/pnas.2209819119. PMC9586297 36215466

[14] O. Yasa , Y. Toshimitsu , M. Y. Michelis , et al., “An Overview of Soft Robotics,” Annual Review of Control, Robotics, and Autonomous Systems 6, no. 1 (2023): 1–29, 10.1146/annurev-control-062322-100607.

[15] C. A. Aubin , B. Gorissen , E. Milana , et al., “Towards Enduring Autonomous Robots via Embodied Energy,” Nature 602, no. 7897 (2022): 393–402, 10.1038/s41586-021-04366-1. 35173338

[16] S. A. Burden , T. Libby , K. Jayaram , S. Sponberg , and J. M. Donelan , “Why Animals Can Outrun Robots,” Science Robotics 9, no. 89 (2024): eadi9754, 10.1126/scirobotics.adi9754. 38657092

[17] J. Veenstra , C. Scheibner , M. Brandenbourger , et al., “Adaptive Locomotion of Active Solids,” Nature 639, no. 8056 (2025): 935–941, 10.1038/s41586-025-08646-3. 40074911

[18] J. Zhou , J. Huang , Q. Dou , P. Abbeel , and Y. Liu , “A Dexterous and Compliant (DexCo) Hand Based on Soft Hydraulic Actuation for Human‐Inspired Fine in‐Hand Manipulation,” IEEE Transactions on Robotics 41 (2024): 666–686, 10.1109/TRO.2024.3508932.

[19] J. Zhou , Y. Chen , Y. Hu , et al., “Adaptive Variable Stiffness Particle Phalange for Robust and Durable Robotic Grasping,” Soft Robotics 7, no. 6 (2020): 743–757, 10.1089/soro.2020.0020. 32319857

[20] J. Zhou , W. Chen , J. Huang , B. Liang , Y. Liu , and M. Tomizuka , “Programmable Locking Cells (PLC) for Modular Robots With High Stiffness Tunability and Morphological Adaptability,” IEEE Transactions on Robotics 41 (2025): 6460–6477, 10.1109/TRO.2025.3626509.

[21] C. H. Belke and J. Paik , “MORI: A Modular Origami Robot,” IEEE/ASME Transactions on Mechatronics 22, no. 5 (2017): 2153–2164, 10.1109/TMECH.2017.2733178.

[22] D. Rus and M. T. Tolley , “Design, Fabrication and Control of Origami Robots,” Nature Reviews Materials 3, no. 6 (2018): 101–112, 10.1038/s41578-018-0009-8.

[23] M. Meloni , J. Cai , Q. Zhang , et al., “Engineering Origami: A Comprehensive Review of Recent Applications, Design Methods, and Tools,” Advanced Science 8, no. 13 (2021): 2000636, 10.1002/advs.202000636.

[24] H. Cao , J. Zhou , K. Chen , Q. He , Q. Dou , and Y. H. Liu , “Design and Optimization of an Origami Gripper for Versatile Grasping and Manipulation,” Advanced Intelligent Systems 6, no. 12 (2024): 2400271, 10.1002/aisy.202400271.

[25] J. Huang , J. Zhou , Z. Wang , et al., “Modular Origami Soft Robot With the Perception of Interaction Force and Body Configuration,” Advanced Intelligent Systems 4, no. 9 (2022): 2200081, 10.1002/aisy.202200081.

[26] Y. Chi , Y. Li , Y. Zhao , Y. Hong , Y. Tang , and J. Yin , “Bistable and Multistable Actuators for Soft Robots: Structures, Materials, and Functionalities,” Advanced Materials 34, no. 19 (2022): 2110384, 10.1002/adma.202110384. 35172026

[27] L. Kong and Y. F. Zhao , “From Origami to Bistable and Laminate Structures: A Review for Multifunctional Applications From Structural Perspective of Shape‐Changing Structures,” Advanced Intelligent Systems 8, no. 2 (2025): 2500505, 10.1002/aisy.202500505.

[28] B. Tan , J. Lyu , F. Yang , et al., “Bistable Origami Thermal Switch With High Switching Ratios,” Nature Communications 17, no. 3177 (2026), 10.1038/s41467-026-69956-2. PMC13046734 41741443

[29] Y. Zhou , M. Ye , C. Hu , H. Qian , B. J. Nelson , and X. Wang , “Stimuli‐Responsive Functional Micro‐/Nanorobots: A Review,” ACS Nano 17, no. 16 (2023): 15254–15276, 10.1021/acsnano.3c03985. 37534824

[30] J. Liu , H. Y. Jiang , J. Q. Tian , et al., “Stimuli‐Responsive Liquid Crystal Elastomers: From Materials to Applications,” Advanced Functional Materials 36, no. 27 (2025): e24606, 10.1002/adfm.202524606.

[31] Z. Shen , F. Chen , X. Zhu , K. T. Yong , and G. Gu , “Stimuli‐Responsive Functional Materials for Soft Robotics,” Journal of Materials Chemistry B 8, no. 39 (2020): 8972–8991, 10.1039/D0TB01412H. 32901646

[32] Y. Zhao , M. Hua , Y. Yan , S. Wu , Y. Alsaid , and X. He , “Stimuli‐Responsive Polymers for Soft Robotics,” Annual Review of Control, Robotics, and Autonomous Systems 5, no. 1 (2022): 515–545, 10.1146/annurev-control-042920-094502.

[33] C. Zhang , Z. Zhang , Y. Peng , et al., “Plug & Play Origami Modules With All‐Purpose Deformation Modes,” Nature Communications 14, no. 1 (2023): 4329, 10.1038/s41467-023-40036-0. PMC10356792 37468465

[34] Z. Zhang , G. Chen , Y. Xun , et al., “Bioinspired Rigid‐Soft Hybrid Origami Actuator With Controllable Versatile Motion and Variable Stiffness,” IEEE Transactions on Robotics 39, no. 6 (2023): 4768–4784, 10.1109/TRO.2023.3311630.

[35] Z. Wang , Z. Wang , Y. Zheng , Q. He , Y. Wang , and S. Cai , “Three‐Dimensional Printing of Functionally Graded Liquid Crystal Elastomer,” Science Advances 6, no. 39 (2020): eabc0034, 10.1126/sciadv.abc0034. 32978149 PMC7518867

[36] Z. Wang , H. Tian , Q. He , and S. Cai , “Reprogrammable, Reprocessible, and Self‐Healable Liquid Crystal Elastomer With Exchangeable Disulfide Bonds,” ACS Applied Materials & Interfaces 9, no. 38 (2017): 33119–33128, 10.1021/acsami.7b10444. 28879760

[37] Q. He , Z. Wang , Y. Wang , et al., “Electrospun Liquid Crystal Elastomer Microfiber Actuator,” Science Robotics 6, no. 57 (2021): eabi9704, 10.1126/scirobotics.abi9704. 34433656

[38] Q. He , Z. Wang , Y. Wang , A. Minori , M. T. Tolley , and S. Cai , “Electrically Controlled Liquid Crystal Elastomer–Based Soft Tubular Actuator With Multimodal Actuation,” Science Advances 5, no. 10 (2019): eaax5746, 10.1126/sciadv.aax5746. 31646178 PMC6788870

[39] Z. Wang , Y. Wang , and H. Zhang , “LEGO‐like Origami Robots Standardize Structure Design of Soft Robots,” Advanced Science 13, no. 5 (2026): e13881, 10.1002/advs.202513881. 41387184 PMC12849906

[40] H. Zhang , “Mechanics Analysis of Functional Origamis Applicable in Biomedical Robots,” IEEE/ASME Transactions on Mechatronics 27, no. 5 (2022): 2589–2599, 10.1109/TMECH.2021.3113030.

[41] J. Zhou , B. Liang , J. Huang , I. Zhang , and M. Tomizuka , “Global‐Local Interface for On‐Demand Teleoperation,” preprint, arXiv, September 25, 2025, arXiv:2502.09960.

