# Supplementary material for: Geometry‐Encoded Actuation as a Structural Interaction Layer in Origami Robots
Source: Adv Sci (Weinh). 2026 Aug 14:e76982. Online ahead of print. doi: 10.1002/advs.76982 (PMC13474188; doi:10.1002/advs.76982)
Supplement: Supplementary file 1 [file ADVS-9999-e76982-s002.pdf]

Supporting Information

Geometry-Encoded Actuation as A Structural Interaction Layer in Origami Robots

Jianshu Zhou*, Yitong Li, Junda Huang, Ian Zhang, Haoran Liu, Boyuan Liang, Yiyuan Zhang, Xiaohui Xiao, Qiguang He*

* Corresponding Authors

Email: jianshuzhou@nus.edu.sg; qiguanghe@cuhk.edu.hk

This PDF file includes:

Materials and Methods

Theoretical analysis

Figures S1 to S9

Tables S1 and S2

Captions for Movies S1 and S2

Other Supplementary Materials for this manuscript include the following:

Movies S1 and S2

Materials and Methods

Materials. (1,4-bis-[4-(3-acryloyloxypropyloxy) benzoyloxy]-2-methylbenzene) (RM257; Shi jia Zhuang Sdyano Fine Chemical Co. Ltd; 95%), 2,2'-(ethylenedioxy) diethanethiol (EDDET; TCI, Shanghai; 95%), pentaerythritol tetrakis (3-mercaptopropionate) (PETMP; TCI, Shanghai, 95%), dipropylamine (DPA; TCI, Shanghai, 98%) and multi-walled carbon nanotubes (MWCNT; Suzhou Tanfeng Graphene Technology Co. Ltd; >99%; Outer diameter: 8-15 nm, Length: 10-50 μm) are used as received without further purification.

Synthesis of LCE-CNT composite film. RM257 (10 g, 17 mmol) and toluene (3.565 g) were mixed in a beaker and heated in an oven at 85°C for about 20 min. Then, the HHMP photoinitiator (0.023 g, 0.1 mmol) was added to the beaker. After the mixture became clear again, MWCNT (0.04 g) was put into the beaker. After that, PETMP (1.529 g, 3.13 mmol) and EDDET (1.902 g, 10.43 mmol) were mixed and subsequently added to the abovementioned mixture. The catalyst PDA (0.032 g, 0.32 mmol) was later poured into the same beaker. The solution was stirred for about 3 min, degassed in a vacuum chamber for about 5 min, and then poured into the glass molds. Next, the molds were placed in a dark environment for 24 hours. In the subsequent step, the cured LCE-CNT film in the molds was placed in the oven at 85°C for 12 hours to evaporate the solvent. After the LCE-CNT film was cooled down to room temperature, it appeared opaque and retained the polydomain state. Last, a monodomain LCE-CNT film was obtained by stretching the tubular to two times its original length under the 365-nm UV irradiation (10 mW cm^{-2}) for 10 min (Figure S1).

Working principle of the electrically controlled LCE actuator

To achieve highly compliant and efficient actuation, a stretchable serpentine metallic heater is embedded within a bilayer structure of liquid crystal elastomer (LCE) thin films (Figure S2-A). This sandwich configuration forms an advanced electrothermal actuator, where the joule heating generated by the serpentine resistor triggers the phase transition of the LCE, leading to rapid, reversible, and controllable macroscopic shape memory effects. When a voltage is applied to the heater, Joule heating is generated, increasing the temperature of the LCE. As the temperature exceeds the nematic-to-isotropic transition temperature, the mesogens become disordered, resulting in a large contraction. This powerful contraction generates a substantial actuation force that effectively lifts an attached payload. After the voltage is removed, the actuator cools down, and the LCE gradually returns to the nematic state, enabling the actuator to recover its original shape (Figure S2-B).

Strain stability of LCE and LCE-CNT films

The cyclic actuation behavior of the pristine LCE (Figure S4-C) and LCE-CNT (Fig.

S4-F) films was evaluated using a hot stage maintained at 130 °C. For each cycle, the film was placed on the hot stage until the thermal contraction stabilized, at which point the equilibrium length was recorded to calculate the actuation strain. Subsequently, the sample was removed from the hot stage to allow complete recovery to its initial length before initiating the next cycle. This procedure was repeated for 100 consecutive cycles to assess the cyclic stability. Thereinto, the actuation strain was defined as $\varepsilon = (\mathrm{L} - \mathrm{l})/\mathrm{L} \times 100\%$, where L and l were the fiber lengths in the initial and deformed states, respectively.

Theoretical Analysis

Statics of Origami Mechanism. This section presents the static mechanical analysis of three representative origami mechanisms. For the derivations, we assume all rods are massless and rigid, hinges are frictionless, and contact friction is negligible. These assumptions allow us to focus on the essential geometric and force–torque relationships governing the mechanisms.

Vertical LCE Mechanism. Applying the law of sines, the geometric constraint is expressed as

$$\sin\alpha = \frac{a}{L}\sin\theta \tag{1}$$

where a and L denote geometric parameters and θ is the actuation angle. Let M denote the torque generated by the origami structure, T the axial force in the LCE, and F the force along rod AB. For the transition from state 1 to state 0, the force–torque relations are given by

$$M = F(b-x)\sin\alpha \tag{2}$$

$$T = 2F\cos\alpha = \frac{2\sqrt{L^2 - a^2\sin^2\theta}}{a(b-x)\sin\theta}M \tag{3}$$

where the factor of 2 arises from the structural symmetry. Simplifying for the vertical mechanism, the LCE force–torque relation becomes

$$\mathrm{T} = \frac{2\sqrt{L^2 - a^2\sin^2\theta}}{ab}\sin\theta\ M \tag{4}$$

Horizontal LCE Mechanism. For the horizontal configuration, applying geometric constraints (case C) yields

$$\cos\alpha = \frac{x-c}{2L_1} \tag{5}$$

$$\tan\alpha = \frac{\sqrt{4L_1^2 - (x-c)^2}}{x-c} \tag{6}$$

where L_1 and c denote geometric parameters and α is the angle of deformation. The lower substructure of the horizontal mechanism is analogous to the vertical mechanism. Incorporating the result from Eq. (4), the LCE force–torque relation is expressed as

$$2T\tan\alpha = \frac{2\sqrt{L_2^2 - a^2\sin^2\theta}}{ax_2\sin\theta}M \tag{7}$$

$$T = \frac{\sqrt{L_2^2 - a^2 \sin^2 \theta}}{a x_2 \tan \alpha \sin \theta} M \tag{8}$$

where L_2 and x_2 denote geometric parameters.

Origami Wheel Mechanism. For the origami wheel, the law of sines provides the geometric relation

$$\frac{a_1}{\sin \alpha} = \frac{x}{\sin \theta} \tag{9}$$

where a_1 is the reference rod length, and x is the effective displacement. From torque balance of the rods, the equilibrium relations are obtained as

$$T \frac{a_2 a_1}{x} \sin \theta = F_1 b \cos \frac{\theta}{2} \tag{10}$$

$$T = \frac{x b F_1}{2 a_1 a_2 \sin \frac{\theta}{2}} \tag{11}$$

where a_2 is the secondary rod length, F_1 is the force transmitted along the rod, and b is the lever arm length.

Energy of Origami Mechanisms. The potential energy patterns of the origami reveal its intrinsic features of bistability. In this section, we will look into the energy modeling of the origami structure. The origami structure is called the Waterbomb Base, which has been in use for centuries. The modeling is carried out by treating the panels as rigid links and the folds as hinges. When symmetric restriction is applied and all folds are biased toward a set of initial angles, the waterbomb base reduces from multiple degrees of freedom to a one degree of freedom device and all link positions can be determined from a single input. Using the formulas of spherical trigonometry, we can calculate the following intermediate variables as shown in the graph.

$$c = \cos^{-1}[\cos^2(\theta)], \tag{12}$$

$$B = \sin^{-1}\left(\frac{\sin(\theta)}{\sin(c)}\right), \tag{13}$$

$$E = \cos^{-1}\left(\frac{\sin(c)}{\cos(c) + 1}\right), \tag{14}$$

where γ_m is the angle of all mountain folds and γ_v is the angle of all valley folds.

$$\gamma_m = \cos^{-1}[2\cos(c) - 1] - 180°, \tag{15}$$

$$\gamma_{va} = 2\,(B + E - 90°). \tag{16}$$

By inserting Eq. (13) and Eq. (14) into Eq. (16), we obtain

$$\gamma_{va} = 2\left\{\sin^{-1}\left(\frac{\sin(\theta)}{\sin(c)}\right) + \cos^{-1}\left(\frac{\sin(c)}{\cos(c)+1}\right) - 90°\right\}. \tag{17}$$

Using different spherical triangles shown in Fig.S5 A, c and γ_m stays the same as defined in Eq. (12) and Eq. (15), but γ_v in Range b, γ_{vb}, is defined as

$$\gamma_{vb} = 2\left\{\cos^{-1}[\csc(c) - \cot(c)] - \sin^{-1}\left(\frac{\sin(\theta)}{\sin(c)}\right) + 90°\right\}. \tag{18}$$

The energy analysis is done by modeling each fold as a compliant small-length flexural pivot with the parameters shown in Fig.S5 A.

The potential energy stored in each pivot is given by

$$V = \frac{1}{2}k\theta^2, \tag{19}$$

where θ denotes the angular deflection from the neutral position, and the stiffness k is defined as

$$k = \frac{EI}{l}, \tag{20}$$

where I is the second moment of area, and l is the length of the flexural pivot. Since the material in the fold came from a flat sheet, the compliant model assumes that the cross section of the fold material remains rectangular and the second moment of area is given as

$$I = \frac{bh^3}{12}. \tag{21}$$

Considering all the folds within the origami (4 mountain folds and 4 valley folds), we obtain the total potential energy

$$V_{TOT} = 2k[(\gamma_m - \gamma_{m0})^2 + (\gamma_v - \gamma_{v0})^2]. \tag{22}$$

Fig.S5 B shows the dimensionless potential energy curve V_{TOT}/k with first stable equilibrium position at $\theta_0 = \pi/3$. It also shows that there is an energy barrier at position $\theta_0 = \pi/2$, which is also the fundamental reason for bistability. The structure needs to receive enough energy to go over the energy barrier, and since the barrier point is also an unstable equilibrium state, the structure will automatically move towards the other stable equilibrium state.

Generally, all mountain folds share the same stiffness k_{γ_m}, and all valley folds share the same stiffness k_{γ_m}, but $k_{\gamma_m} \neq k_{\gamma_v}$, so the total potential energy becomes

$$V_{TOT} = 2\left[k_{\gamma_m}(\gamma_m - \gamma_{m0})^2 + k_{\gamma_v}(\gamma_v - \gamma_{v0})^2\right]. \tag{23}$$

Fig.2C shows the nondimensionalized potential energy curve V_{TOT}/k_{γ_m} with different stiffness ratio $k_{\gamma_v}/k_{\gamma_m}$. By Eq. (20) and Eq. (21), we know that $k \propto b$, where b is the width of the compliant material. In the square waterbomb origami structure shown in section 2.1, the stiffness ratio is

$$k_{\gamma_v}/k_{\gamma_m} = 1/\sqrt{2}. \tag{24}$$

Using the principle of virtual work, we can derive the torque generated by the origami with

$$M = \frac{dV_{TOT}}{d\theta}. \tag{25}$$

Fig.S5 C shows the relationship between the dimensionless torque and θ. The figure shows that the torque values exhibit good linearity around the two stable equilibrium points, while the simulation indicates a detailed energy distribution during the origami transition (Fig. S6 A and S6 B).

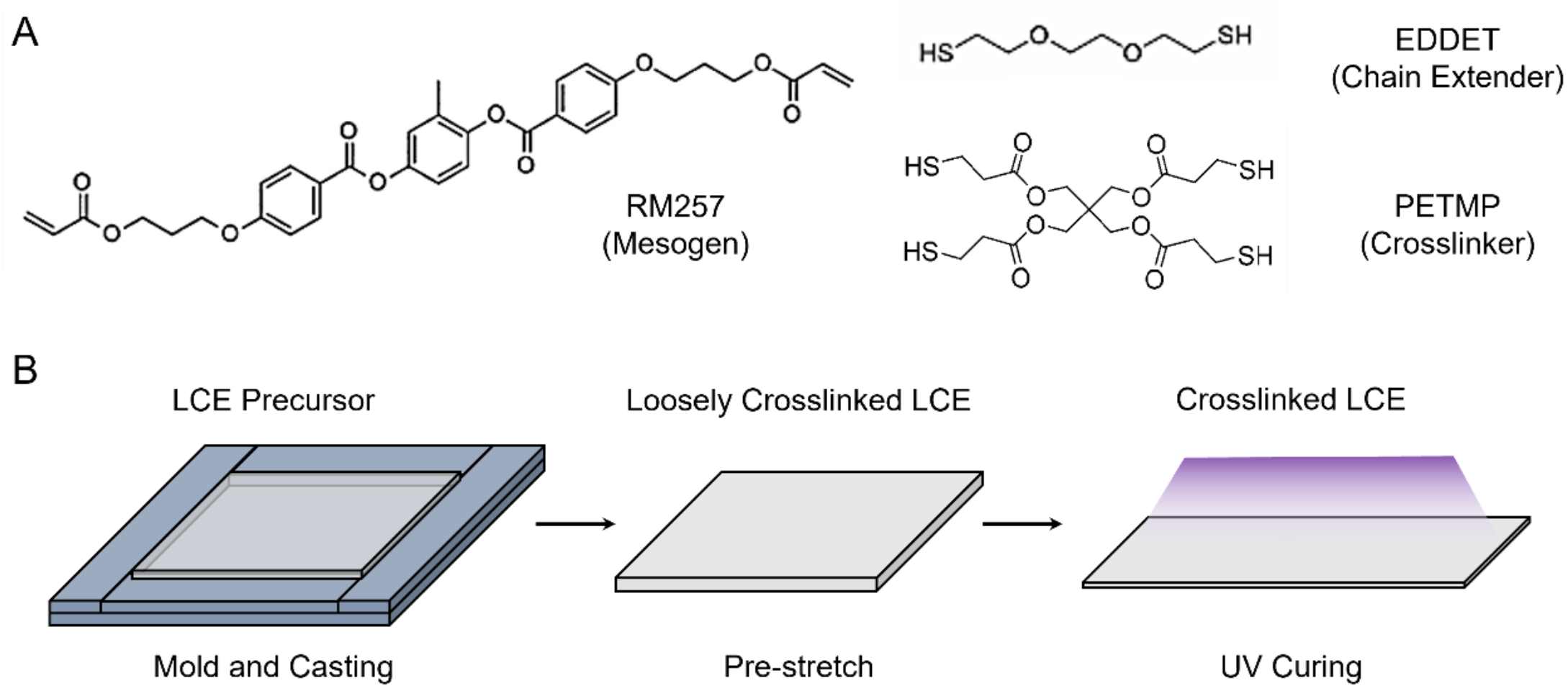

Figure S1. Fabrication process of the liquid crystal elastomer (LCE). (A) Chemical structures of the components used for LCE synthesis. RM257 serves as the liquid-crystal mesogen, EDDET acts as the chain extender, and PETMP functions as the cross-linker. (B) Schematic illustration of the LCE fabrication process. First, the LCE precursor was poured into a mold and cured to form a loosely cross-linked network. The partially cross-linked LCE film was then removed from the mold and stretched to align the mesogen. Finally, the aligned LCE was exposed to UV light for secondary curing to fix the mesogen orientation.

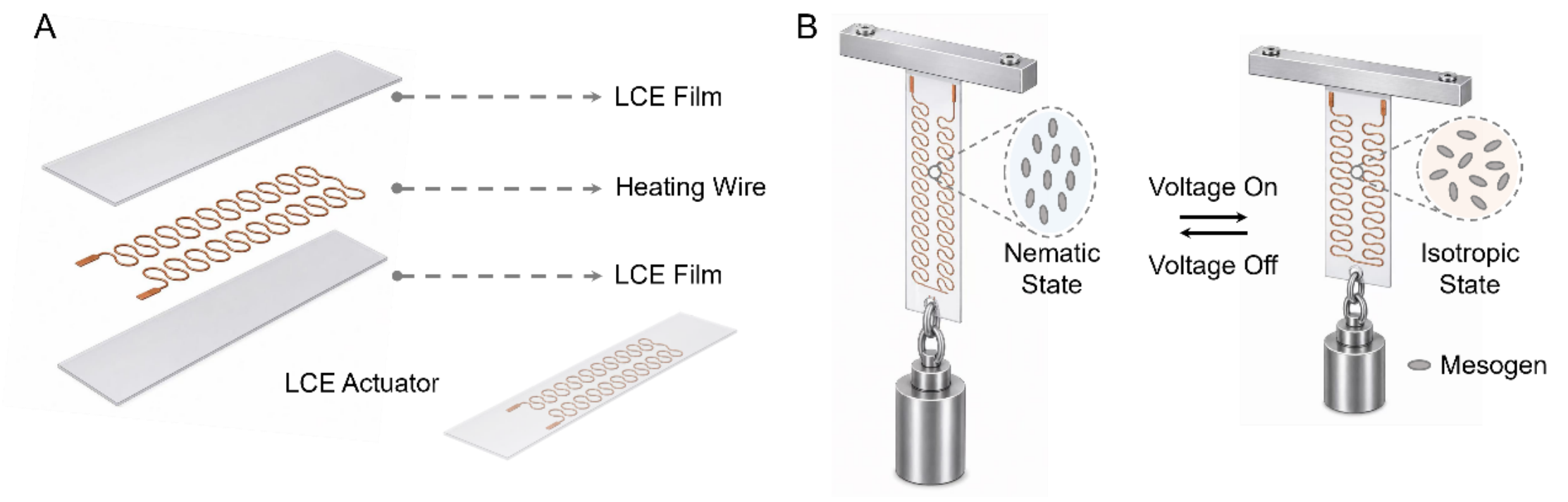

Figure S2. Design and operating principle of the electrically controlled LCE actuator. (A) Structure of the LCE actuator. A stretchable serpentine heater is sandwiched between two LCE thin films, forming an electrothermal actuator. (B) Operating principle of the actuator. When a voltage is applied to the heater, Joule heating is generated, increasing the temperature of the LCE. As the temperature exceeds the nematic-to-isotropic transition temperature, the mesogens become disordered, resulting in a large contraction. After the voltage is removed, the actuator cools down, and the LCE gradually returns to the nematic state, enabling the actuator to recover its original shape.

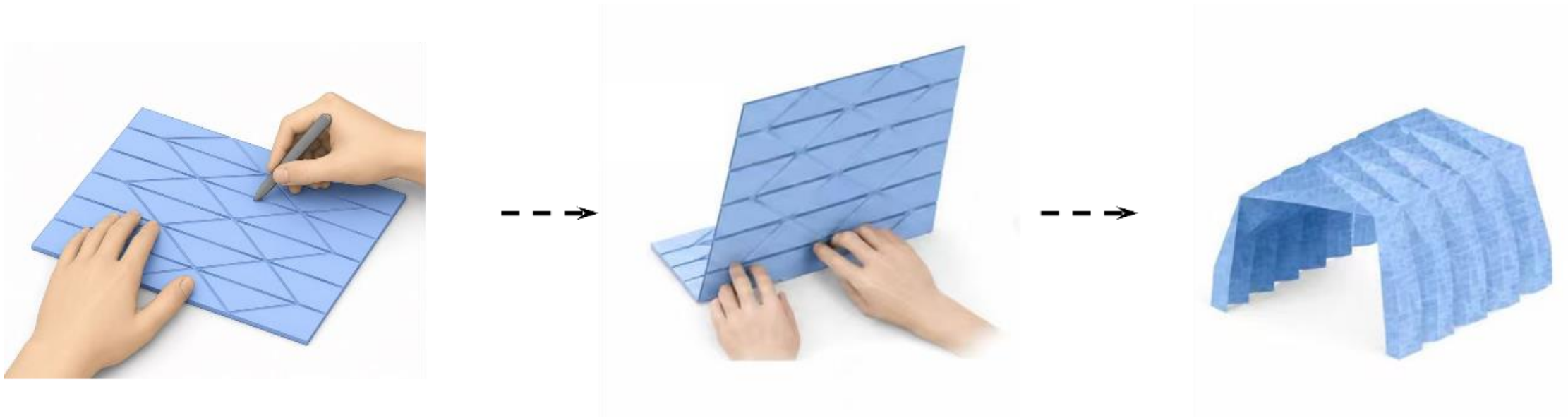

Figure S3. Fabrication of the origami structure. Crease patterns were first generated on a flat sheet using a razor blade or a craft knife. The patterned sheet was then folded along the predefined mountain and valley folds to form the final three-dimensional origami structure.

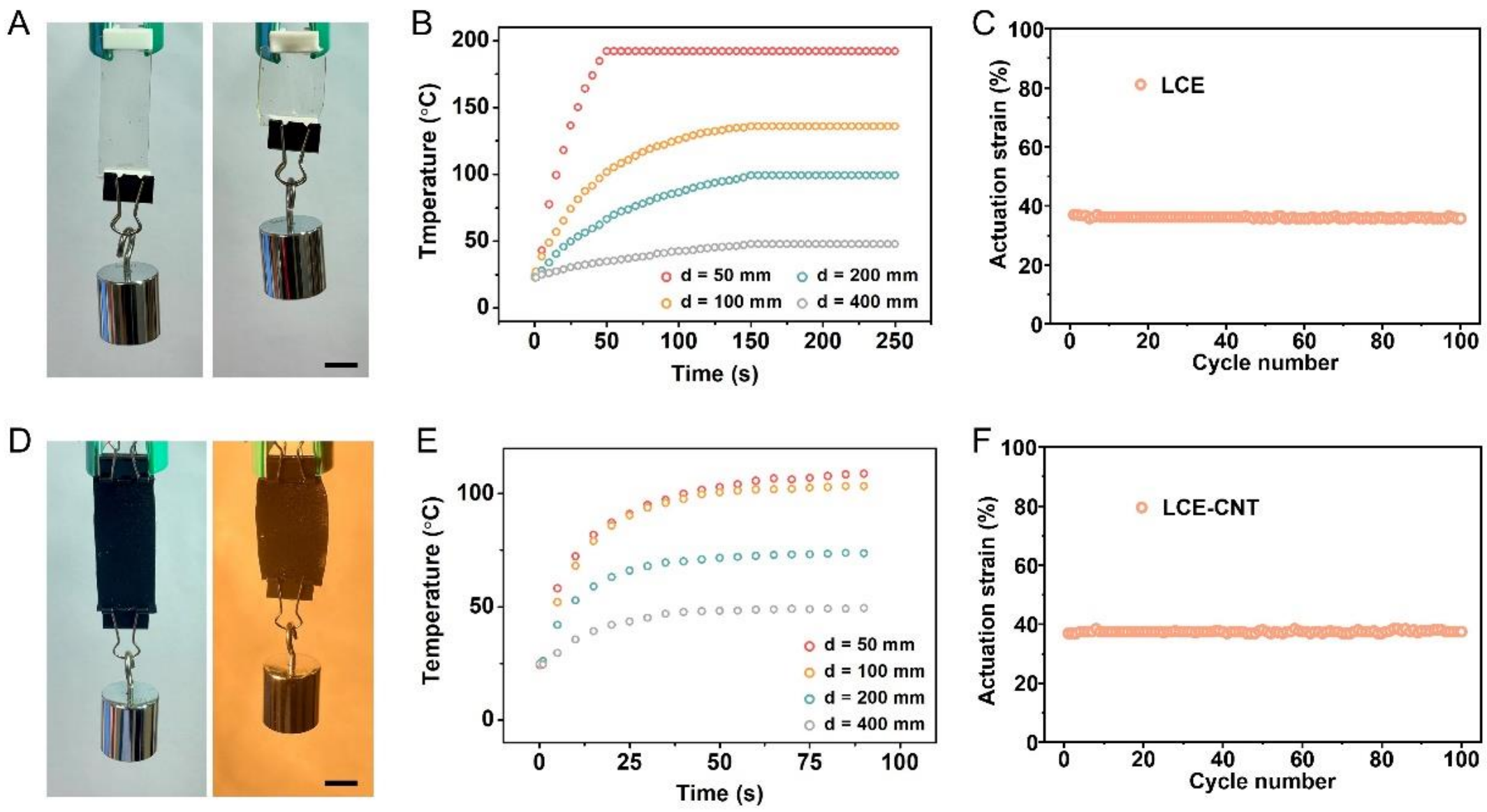

Figure S4. Characterization of pristine LCE and CNT-LCE. (A) Images of the pristine LCE before and during thermal actuation, showing large contraction upon heating. (B) Surface temperature as a function of time under different distances between the heat source and the LCE. A shorter distance results in a higher equilibrium temperature. (C) Durability test of the pristine LCE actuator over 100 actuation cycles, demonstrating stable performance without noticeable degradation. (D) Images of the CNT-LCE before and during light-induced actuation, showing significant contraction under light illumination. (E) Surface temperature as a function of time for the CNT-LCE under different light-source distances, where a shorter distance leads to a higher surface temperature. (F) Durability test of the CNT-LCE actuator under repeated light irradiation, showing stable actuation performance over 100 cycles. Scale bar in A and D: 20 mm.

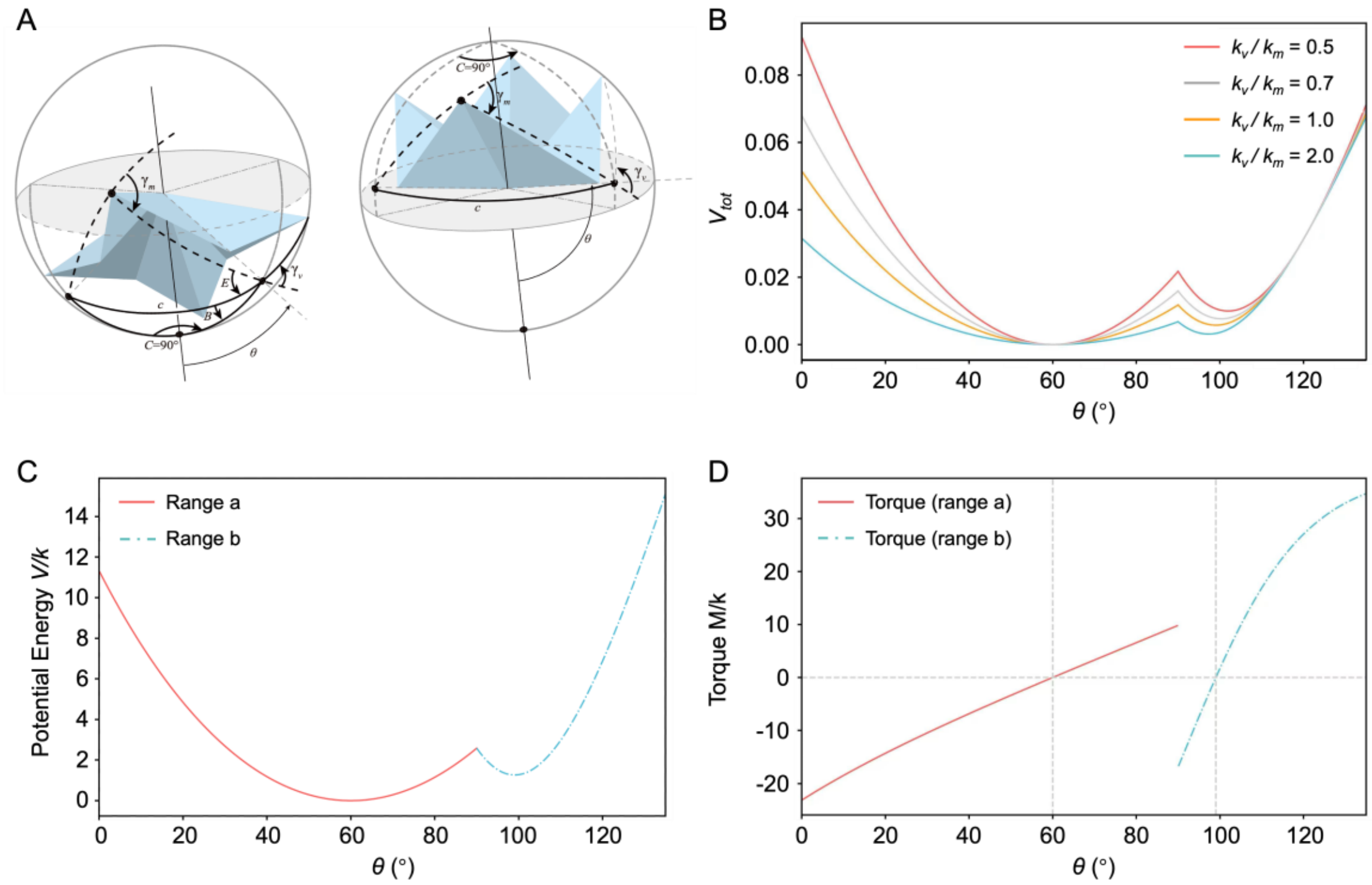

Figure S5. Mechanism demonstrations. (A) Geometric analysis of spherical triangles used to calculate the dihedral angles γm and γv over different folding ranges. (B) Dimensionless potential energy curves, V_{TOT}/k, under different stiffness ratios $k_{\gamma v}/k_{\gamma m}$. (C) and (D) Dimensionless potential energy, V_{TOT}/k (top), and normalized torque, M/k (bottom), with equilibrium positions highlighted for Range a ($\theta \in [0°, 90°]$) and Range b ($\theta \in [90°, 135°]$).

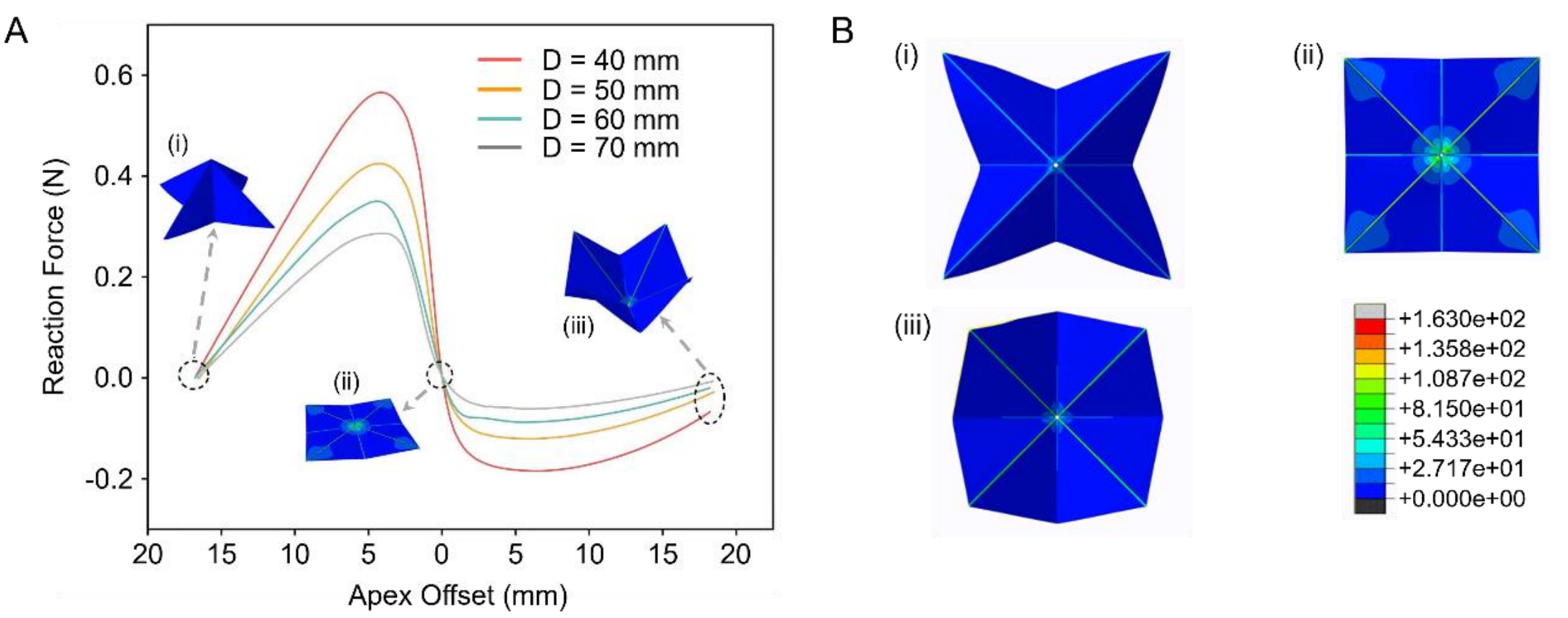

Figure S6. Evolution of the folding configuration and corresponding energy landscape during the transition between the two equilibrium states. (A) Experimental validation of the predicted bistable behavior, showing reversible switching between the folded and deployed configurations. (B) Comparison between theoretical predictions and experimental measurements of the equilibrium angle and restoring torque.

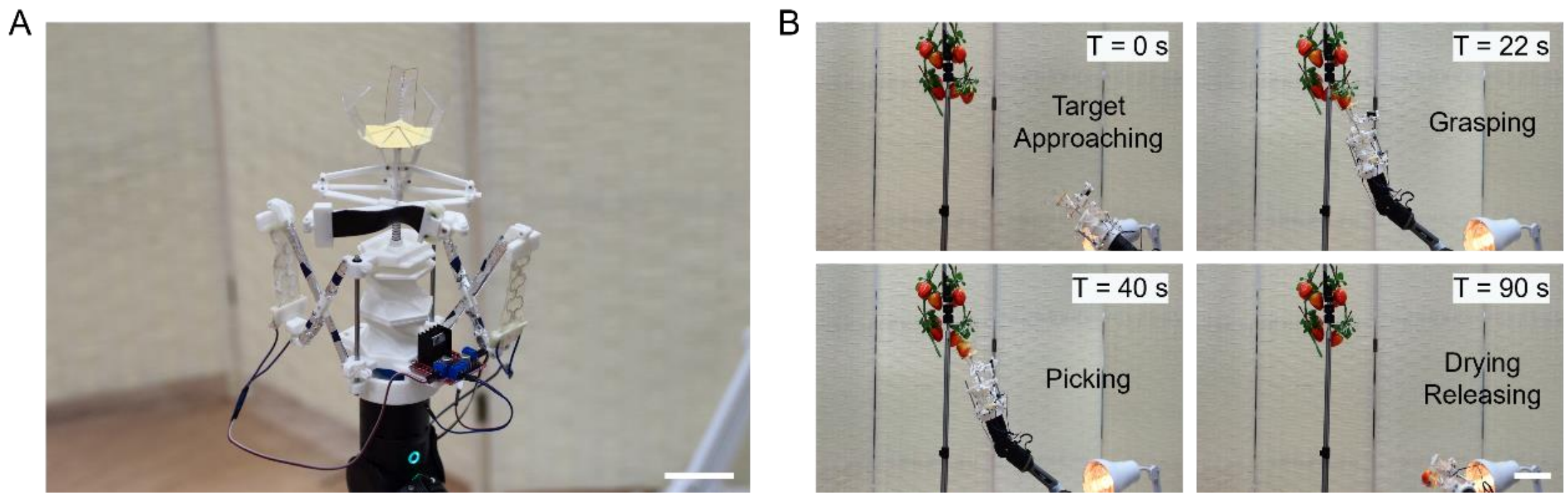

Figure S7. Autonomous origami robotic arm for fruit harvesting. (A) Photograph of the GEA enabled manipulation system, consisting of an origami gripper and an origami robotic wrist. (B) Schematic illustration of the autonomous harvesting process. First, the robotic arm is guided by a mechatronic system to approach the target object (strawberry). Upon contact with the target, the gripper automatically closes through GEA, enabling grasping without additional sensing or control. Subsequently, the electrically controlled LCE actuator is triggered, inducing origami twisting of the robotic arm and generating the motion required for fruit picking. After harvesting, the robotic wrist transports the fruit to the drying area. Exposure to the elevated temperature in the drying environment causes the gripper to reopen, resulting in autonomous object release and completion of the harvesting cycle. Scale bars in A and B: 50 mm.

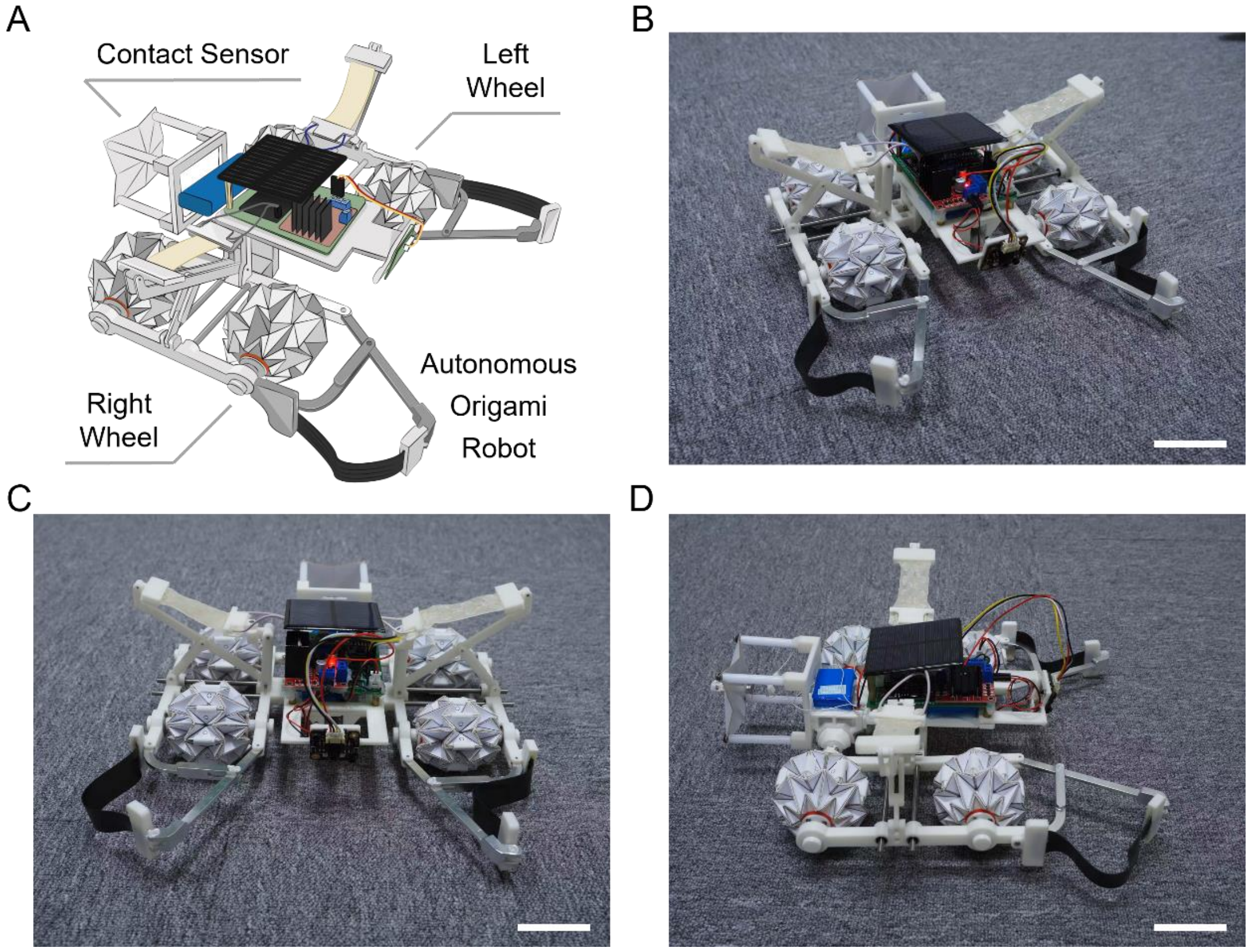

Figure S8. Autonomous origami robot for locomotion. (A) Schematic illustration of the autonomous origami robot, consisting of a contact sensor, a left wheel, and a right wheel. (B–D) Photographs of the robot from different perspectives. Scale bars in B, C, and D: 50 mm.

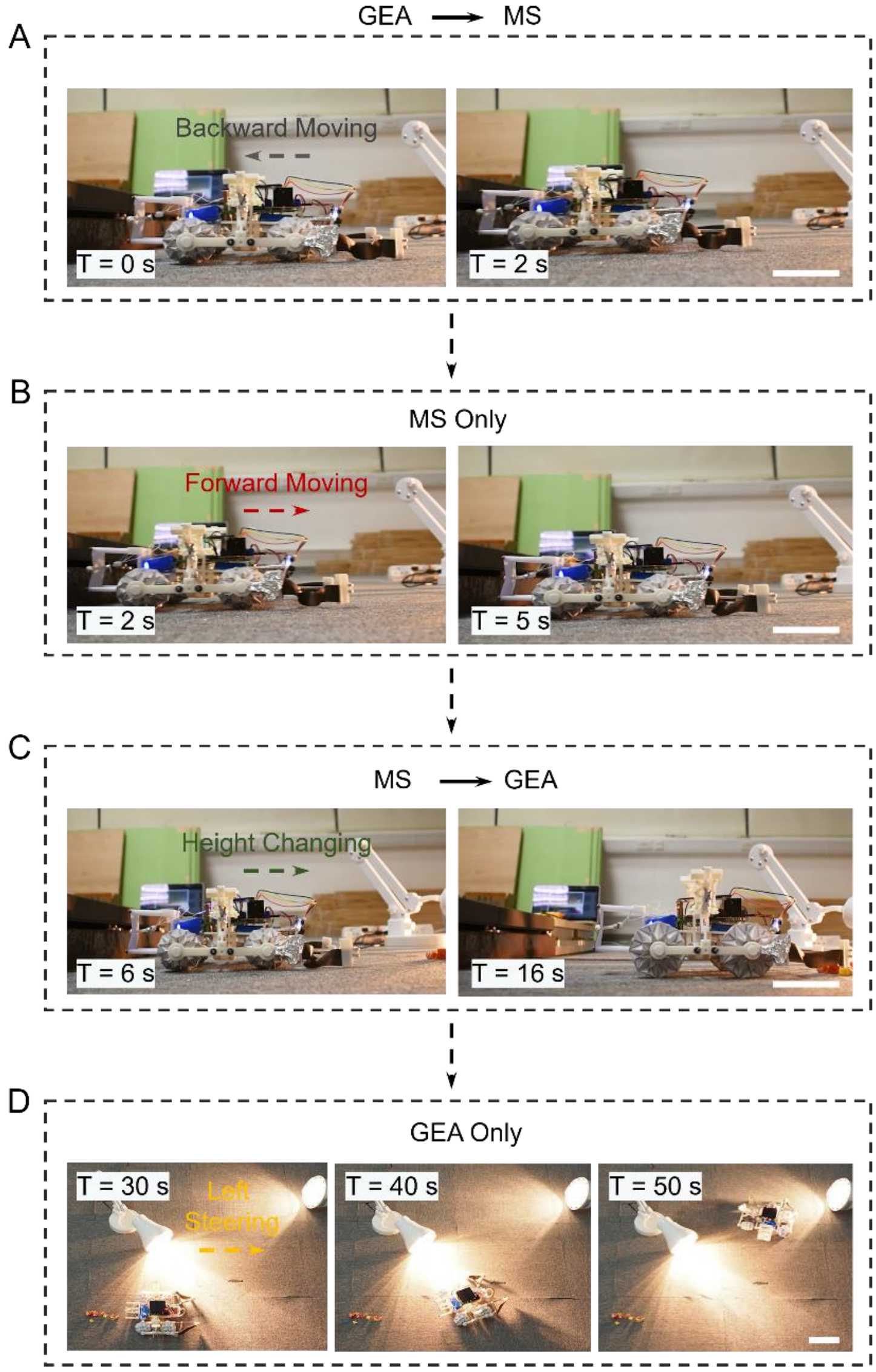

Figure S9. Autonomous locomotion behaviors of the origami robot. (A) Autonomous reversal of locomotion direction. The robot initially moves backward. Upon contacting a wall, the contact sensor activates the GEA, which triggers the conventional mechatronic system and reverses the robot's moving direction. (B) Conventional mechatronic locomotion control, where the robot moves forward under programmed control. (C) Autonomous obstacle-crossing behavior. When the robot encounters an obstacle, the mechatronic system (MS) sends a signal to the GEA, triggering the expansion of the origami wheels. The increased wheel diameter enables the robot to overcome obstacles. (D) Autonomous light-seeking behavior enabled solely by GEA. The robot automatically steers toward the light source without electronic sensing, computation, or feedback control. Scale bars in (A–D), 50 mm.

Table S1. Fatigue characterization of the Waterbomb Origami Gripper during cyclic operation

Number of Cycles	Bistable Switching Functionality	Structural Integrity
1	Functional	Intact
250	Functional	Intact
500	Functional	Intact
750	Functional	Slight softening observed
1000	Functional	Slight softening observed

Table S2. Fatigue characterization of the Origami Wheel during cyclic operation

Number of Cycles	Expansion–Contraction Functionality	Structural Integrity
1	Functional	Intact
250	Functional	Intact
500	Functional	Slight softening observed
750	Functional	Moderate softening observed
1000	Functional	Minor fatigue damage at crease regions

Movie S1. Demonstration of autonomous reaching, gripping, picking, transporting, and releasing enabled by the origami robotic arm.

Movie S2. Demonstration of autonomous backward locomotion, forward locomotion, wheel expansion for obstacle crossing, and light-guided steering of the origami robot.
